# Testicular somatic and germ cell maturation during rhesus macaque development

**DOI:** 10.1073/pnas.2419995122

**Published:** 2025-06-26

**Authors:** Enrique Sosa, Sissy E. Wamaitha, Fei-man Hsu, Mary Jasmine D. Lara, Kiana Oyama, Maggie Custer, Melinda Murphy, Jon D. Hennebold, Young Sun Hwang, Amander T. Clark

**Affiliations:** ^a^Department of Molecular, Cell and Developmental Biology, University of California Los Angeles, Los Angeles, CA 90095; ^b^Eli and Edythe Broad Center of Regenerative Medicine and Stem Cell Research, University of California Los Angeles, Los Angeles, CA 90095; ^c^Molecular Biology Institute; University of California Los Angeles, Los Angeles, CA 90095; ^d^Division of Reproductive and Developmental Sciences, Oregon National Primate Research Center, Beaverton, OR 97006; ^e^Department of Obstetrics and Gynecology, Oregon Health and Science University, Portland, OR 97239

**Keywords:** testicular maturation, gonadal development, rhesus macaque, single-cell RNA-seq

## Abstract

The formation of bilateral testes in animals is critical for puberty, reproductive capacity, and testosterone production across the life course. In humans, testis development begins in embryonic life in the first trimester, with considerable effort focused on the cell and developmental events associated with testis cell specification, leaving limited knowledge on testicular organogenesis during the second and third trimesters. To fill this knowledge gap, we evaluated testicular cell maturation at weeks 5 (W5), W6, W8, W15, and W19 postconception using a rhesus macaque model. Our data identify a major transcriptional change in the somatic cells of the testis (Sertoli cells, interstitial cells and fetal Leydig cells) between W8 and W15, and this is associated with the maturation of seminiferous cords and maturation of PGCs into fetal spermatogonia. Through this work, we identified cellular changes and differential protein expression between W5 and W19 that can be used to holistically define testis development across the time course of embryonic and fetal life. This study provides important insights necessary to recreate the testicular niche from stem cells for biomedical research.

## Significance

This study provides crucial insights into testicular development by examining cell maturation during gestation in the rhesus macaque. The findings reveal significant transcriptomic changes in Sertoli and interstitial fibroblasts after sex determination, linked to the maturation of seminiferous cords and primordial germ cells. Identifying specific markers for Sertoli cells, interstitial fibroblasts, and germ cells enhances our understanding of testis development. These insights are vital for recreating the testicular niche from stem cells, advancing biomedical research as well as their therapeutic application in treating infertility.

The formation of internal and external sex organs before birth, the capacity to progress through puberty, the ability to produce sperm and conceive a biological child, and the production of testosterone over the life course require the formation of reproductive organs called testicles. In human embryonic development, the testis begins to form at around week 5 postconception (wpc, W) with the formation of bilateral gonadal ridges (GRs) located on the medial-lateral side of embryonic kidneys called mesonephros. Each GR is colonized by primordial germ cells (PGCs) that originated three weeks earlier from their site of specification in the posterior amnion of primates, having migrated through the yolk sac and dorsal mesentery to colonize the GR at week 4 to 5 (1). Around the time that PGCs reach the GR, expression of Sex-Determining Region Y (SRY) in bipotential early supporting gonadal cells [ESGCs (1)] initiates a sex-specific transcriptional network to specify the supporting Sertoli cells of the testis from GR cells (2). These newly specified Sertoli cells express the transcription factor SOX9, which is critical for reinforcing and maintaining Sertoli cell fate and establishing the epithelial seminiferous cords. Seminiferous cords are lined by laminin basement membranes that are continuous with the rete testis epithelium developing at the gonadal–mesonephros junction. Anti-Müllerian Hormone (AMH) is expressed by the Sertoli cells of the cords by the 8 W (3). This fetal hormone is responsible for regressing the Mullerian ducts that develop into the female reproductive tract (4). At around the same time, testosterone is produced by the fetal Leydig cells in the testicular interstitium. The fetal testosterone enters the circulation to bind androgen receptors, thus establishing testosterone programming during fetal life (5).

In the second trimester, at W12–17, major cell-fate changes occur in the germline where PGCs initiate differentiation into fetal-stage spermatogonia called postmitotic T1 and T2 Spermatogonia, collectively known also as fetal state 0 spermatogonia (6–8). After this point of development, the field has limited knowledge of testicular formation in the third trimester except for the physiological phenomenon of bilateral descent and seminiferous cord changes during the late fetal period (9, 10). At birth, the human testis is enveloped in a fibrous capsule called a tunica albuginea and filled with seminiferous cords composed of Sertoli cells and spermatogonia. Outside the cords, the major cell types include blood vessels, nerves, lymphatics, testicular fibroblasts, and fetal Leydig cells, which are embedded in an extracellular matrix of Laminin, Fibronectin, and Collagens (11, 12).

Due to the difficulty in obtaining human samples prior to 5-wk, researchers have used primates, most notably the cynomolgus (cyno) macaques, as models. Early single-cell RNA sequencing (scRNA-seq) studies of cyno used embryo samples spanning PGC specification (E13–E20) and sex determination (E36–E55), which collectively have advanced our understanding of early PGC and gonadal development (13–15). However, testicular programming of germline and somatic cells in the later-half of pregnancy remains largely unexplored, yet, this is a period of growth critical for the proper development of the testis. In the current study, we trace testicular development using a different species of macaque, the rhesus macaque (*Macaca mulatta*). Pregnancy in the rhesus macaque is approximately 168 d (24 wk) (16). The rhesus macaque, herein referred to as rhesus, is not considered an endangered species (IUCN 2023), the genome has been sequenced (17), pregnancies are generally singletons (18), there is no obvious diapause (19), young males will progress through puberty (20) and fertility extends across the adult animal’s lifespan (21) making it an ideal model for human testicular biology.

## Results

### Cellular Changes Occur Between W8 and W15 in the Developing Fetal Testis.

**Fig. 1.**
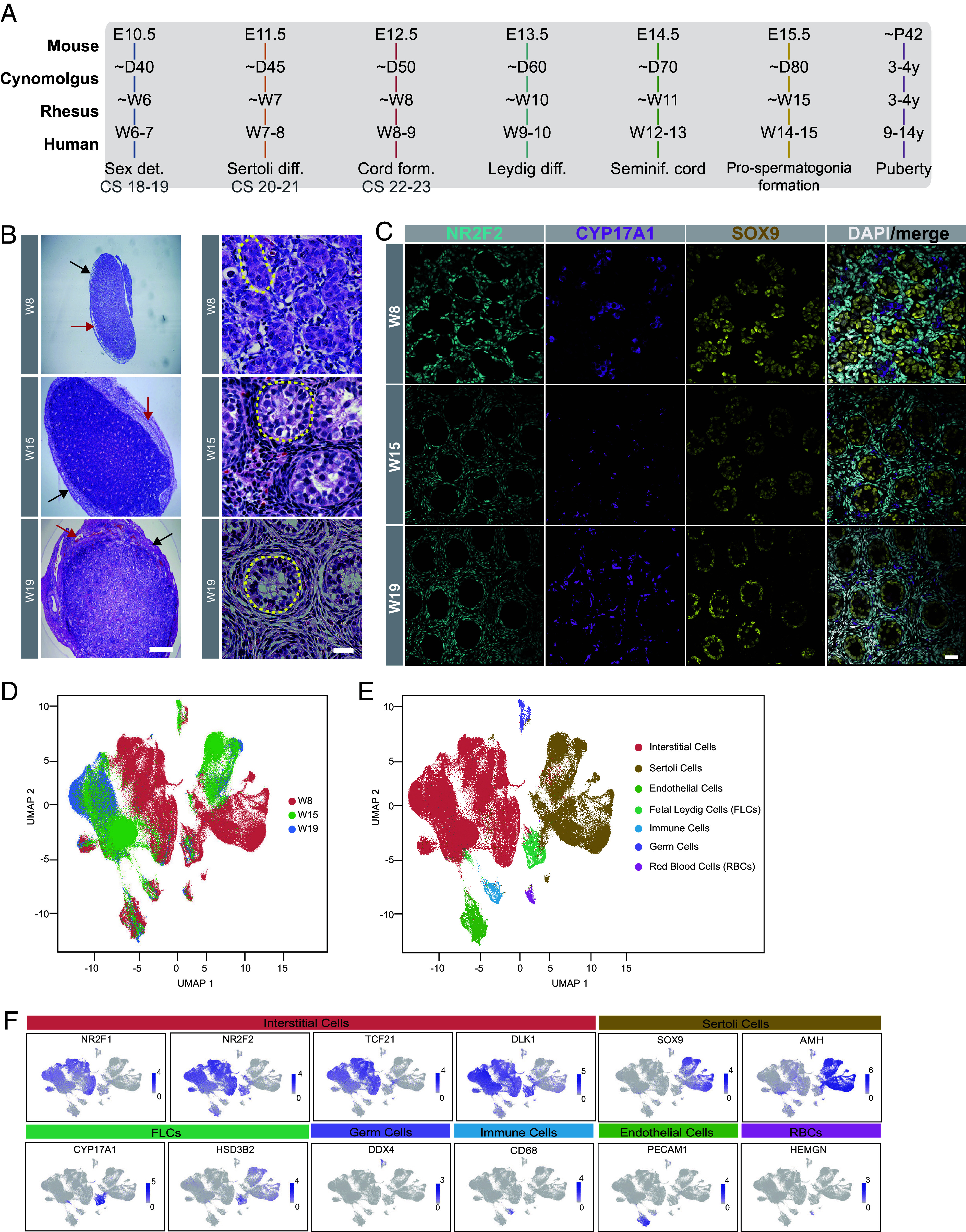
Testis development and single-cell RNA-seq in the rhesus macaque in the first, second, and third trimesters. (*A*) Illustration comparing the timing of selected testicular development events identified in the mouse, primates (cynomolgus or rhesus macaque), and human studies. (*B*) *Left*, Hematoxylin and Eosin (H&E) staining of embryonic and fetal testes at W8, W15, and W19. Black arrows indicate the tunica albuginea. Red arrows indicate the location of blood vessels in the tunica albuginea. (Scale bar, 100 µm); *Right*, High magnification images of (*B*) *Left*. Yellow dotted circles highlight the cord (W8) and tubule structures (W15, W19). (Scale bar, 20 µm). (*C*) Immunofluorescence staining of the testicles at time points indicated showing interstitial fibroblasts stained for NR2F2 (cyan), fetal Leydig cells stained for CYP17A1 (magenta) and Sertoli cells stained for SOX9 (yellow). Nuclei (gray) were stained with DAPI. (Scale bar, 20 µm.) (*D*) UMAP plot of 227,944 testicular cells revealing distinct clustering of W8 testis samples (red) relative to W15 (green) and W19 (blue) samples. (*E*) UMAP plot from (*D*) annotated with testicular cell identities reveals that Sertoli cells and interstitial cells of the rhesus testis exhibit major transcriptional differences at W8 compared to W15 and W19. Other cell types including fetal Leydig cells, endothelial cells, and immune cells and germ cells do not exhibit this type of major transcriptional shift with time (*F*) UMAP feature plots displaying marker gene expression for the annotation of testicular cell types identified in (*E*). Diagnostic genes include interstitial cells (NR2F1, NR2F1, TCF21m and DLK1), Sertoli cells (SOX9 & AMH), fetal Leydig cells (CYP17A1 and HSD3B2), germ cells (DDX4), immune cells (CD68), endothelial cells (PECAM1), and red blood cells (HEMGN). The total number of samples displayed include n = 5 biological replicates at W8, n = 4 biological replicates at W15 and n = 3 biological replicates at W19.

To begin the study, we focused on collecting biological replicates at day week 8 postconception (W8); see *SI Appendix*, Fig. S1*A*; the time of testis cord formation), W15 (pro-spermatogonia formation), and W19 (one month before birth) to identify testicular niche changes as the germline differentiates from PGCs into pro-spermatogonia (Fig. 1*A*). Based on the estrogen peak measurement of each adult female, time-mated breeding was performed (*SI Appendix*, Fig. S1*B*). Genotyping for XY cell-free DNA was performed on the serum of the pregnant animal so that only XY embryos/fetuses were used in the analysis (*SI Appendix*, Table S1). To obtain embryonic and fetal samples, C-sections were performed at the indicated times to remove the conceptus. To confirm the success of embryo staging, we measured embryo weights (*SI Appendix*, Fig. S1*C*) and crown-rump lengths (*SI Appendix*, Fig. S1*D*), which reveals limited variability between biological replicates. Significant differences in weights and lengths were identified when comparing embryos/fetuses at W8, W15, and W19 (*SI Appendix*, Fig. S1 *C* and *D*). Both testicles were removed from embryos at W8, and fetuses at W15 and W19 (*SI Appendix*, Fig. S1*E*). The design of our study involved one testis being fixed in 4% paraformaldehyde for immunofluorescence and the other processed to a single cell suspension for single cell RNA sequencing (scRNA-seq) using the 10X Genomics platform. Tissue was also collected to confirm genotypic sex.

To evaluate the morphology of the embryonic and fetal testis, we first performed hematoxylin and eosin (H&E) staining (Fig. 1*B*) and immunofluorescence (IF) for the ECM and basement membrane protein Laminin (*SI Appendix*, Fig. S1*F*) to examine testis architecture. H&E staining at W8 revealed a simple capsule called the tunica albuginea (Fig. 1*B*, black arrows), which, in addition to the stroma, contains clearly defined blood vessels (Fig. 1*B*, red arrows). At W15 and W19 the tunica albuginea became larger and more complex, with a rich network of coelomic vessels. At higher power, the seminiferous cords are visible in the embryonic testis W8 (an example outlined in yellow hash lines), and by W19, these cords progress to seminiferous cords with evidence of nascent openings. During cord maturation, the cells of the interstitium also change from simple embryonic mesenchyme at W8 to a more complex niche with oriented fibroblast-like cells around the seminiferous cords at W19 (Fig. 1*B*). This result indicates that testis development progresses as a continuum from W8 to W19.

To confirm the identity of major somatic cell types, we performed immunofluorescence for NR2F2 (interstitial fibroblast marker), CYP17A1 (Leydig cell marker), and SOX9 (Sertoli cell marker) and showed that all three proteins are expressed across time with interstitial cells and Leydig cells in the interstitium of the developing testis, and Sertoli cells closely associated in cords. Notably, at W8 the SOX9+ Sertoli cell nuclei fill the cords, whereas by W19, SOX9+ nuclei are primarily localized to the outer edge of the cords, likely closely associated with the basement membrane (Fig. 1*C*). To evaluate basement membrane formation, we performed immunofluorescence for Laminin (*SI Appendix*, Fig. S1*F*) and show Laminin deposition around the cords starting from W8. Laminin staining is also observed in the interstitial spaces at all three time points. Taken together, by W8, the basic architecture of the testis is established, with notable morphological changes to the seminiferous cords, interstitium, and tunica albuginea occurring by the third trimester.

### Identification of Seven Major Cell Types in the Rhesus Macaque Testis Using Single Cell RNA-Seq.

To identify major cell types of the developing rhesus macaque testis, we performed scRNA-seq using the 10X Genomics platform at W8 (N = 5), W15 (N = 4), and W19 (N = 3) (Fig. 1*D*). Testicles were dissociated to a single cell suspension (22), and 10X Genomics was performed in technical duplicate (A and B) or technical triplicate (A, B, and C) (*SI Appendix*, Table S1). For two of the samples (W8_5 and W15_4), we performed a tubule enrichment protocol to facilitate the enrichment of the germ cells. Our initial analysis found no difference between technical replicates within each sample. Therefore, the reads for each technical replicate were combined to generate the total reads in each biological sample (*SI Appendix*, Table S2).

To identify the major cell types of the testis, we performed dimensionality reduction and generated a UMAP of all biological samples (n = 12 samples total), resulting in 18 clusters (*SI Appendix*, Fig. S2 *A* and *B*). Using diagnostic genes, the 18 clusters were found to represent 7 major cell types (Fig. 1 *E* and *F*). These include interstitial fibroblasts (*NR2F1, NR2F2*, *TCF21*, and *DLK1),* Sertoli cells (*SOX9, AMH*), fetal Leydig cells (*CYP17A1/HSD3B2*), germ cells (*DDX4*), endothelial cells (*PECAM*), immune cells (*CD68*), and red blood cells (*HEMGN*). As predicted, due to the rare nature of germ cells in the dataset, most germ cells originated from the tubule-enriched samples at W8 and W15 (*SI Appendix*, Fig. S2 *A*–*D*). DDX4-positive germ cells express other germ cell markers such as *TFAP2C*, *DND1,* and *NANOS3* at all stages, but *PIWIL4* was expressed exclusively in later germ cells at W15 and W19 (*SI Appendix*, Fig. S2*E*).

By examining the proportions of cells in each sample, the dominant cell types identified in the 10X single cell RNA-seq dataset are interstitial cells and Sertoli cells, with interstitial cells making up around 50% or more of sequenced cells in each biological sample (*SI Appendix*, Fig. S2 *C* and *D*). Analysis of the interstitial cells in UMAP indicates that interstitial cells at W8 cluster separately from interstitial cells at W15 and W19, suggesting that a major transcriptional shift in cell identity occurs at this time. Similarly, Sertoli cells also exhibited a similar trend, with W8 Sertoli cells forming a distinct group from the Sertoli cells identified at W15 and W19. As expected, the tubule-enriched samples exhibited higher proportions of Sertoli cells and germ cells relative to nonenriched samples. However, a large fraction of interstitial cells was still detected (*SI Appendix*, Fig. S2*D*). Notably, the endothelial cells, and immune cells at the three time points each formed their own clusters. In addition, although fetal Leydig cells also formed their own cluster, a small group of cells could also be seen with the interstitial group consistent with their interstitial origin. Therefore, the major transcriptional shift observed by Sertoli cells, interstitial fibroblasts, and possibly fetal Leydig cells appears to be unique to these cell types in the developing testis. Taken together, by designing the 10X experiment with biological replicates at W8, W15, and W19, we were able to pinpoint a major transcriptional shift in Sertoli cells and interstitial fibroblasts between W8 and W15 of rhesus macaque testicular development.

### Progression of Sertoli Cells Between W8-W19 Is Associated with Cord Maturation.

**Fig. 2.**
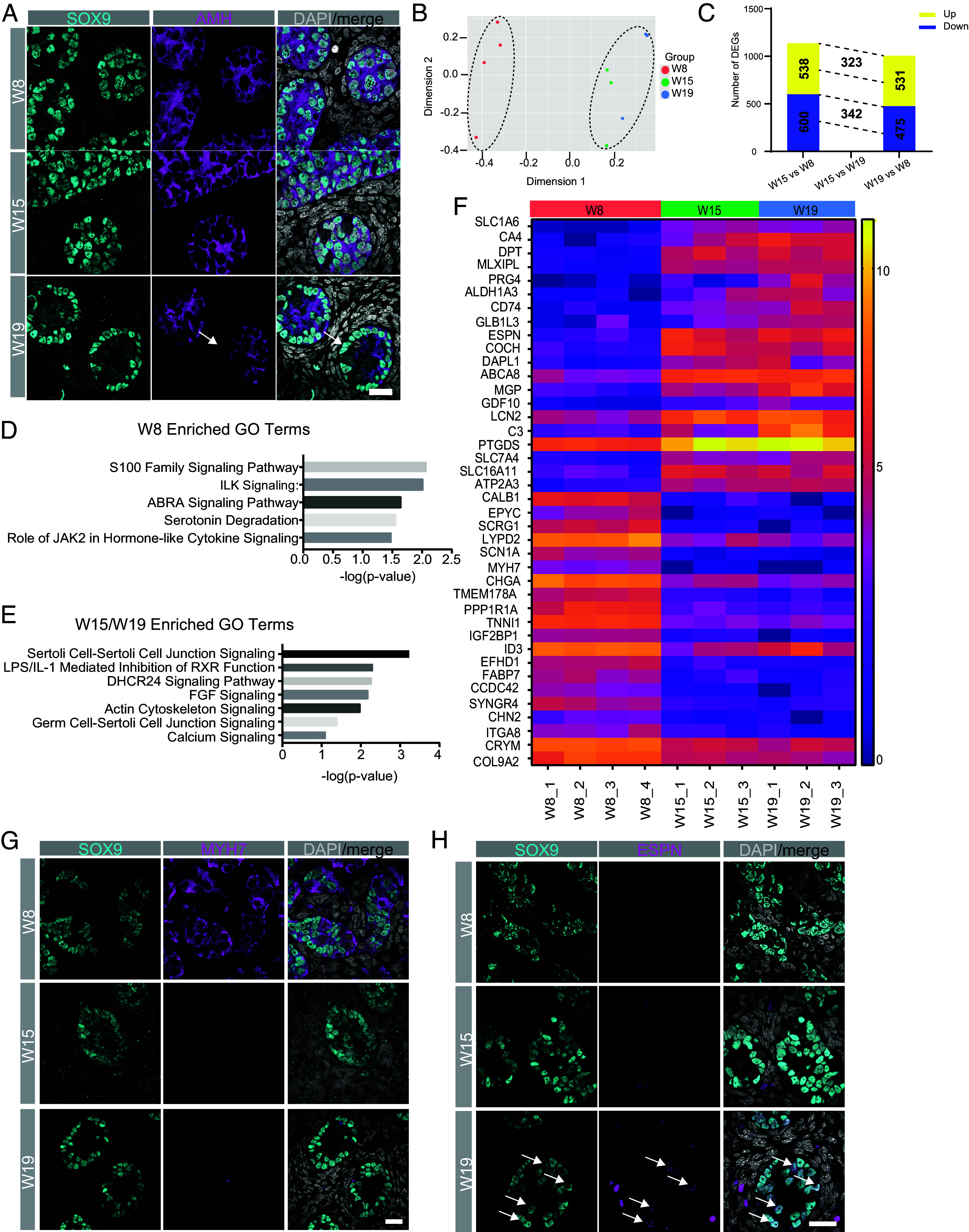
Sertoli cell identity matures between the first and second trimester. (*A*) IF of diagnostic Sertoli cell markers SOX9 (Cyan) and AMH (magenta) reveals stable expression in Sertoli cell cords across the three trimesters. White arrows indicate AMH-negative Sertoli cells at W19. (*B*) Unsupervised Multidimensional Scaling (MDS) plot of SOX9+ AMH+ Sertoli cell clusters from Fig. 1*D*. Each dot represents a biological replicate. W8 (red) n = 4; W15 (green) n = 4; W19 (blue) n = 3. (*C*) Bar graph of the number of differentially expressed genes (DEG) that are either up-regulated (yellow) or down-regulated (purple) between the compared groups as shown (FDR adjusted *P* value <0.05 log2 Fold change > 1). The number of DEGs is shown on the bar. The number of overlapping DEGs between comparisons is shown between the dotted lines. (*D* and *E*) Ingenuity Pathway Analysis (IPA) highlighting pathways enriched in W8 Sertoli cells but repressed in W15 and W19 (*D*) and pathways that become enriched in Sertoli cells as they mature from W8 to W15 and W19 (*E*). (*F*) Heat Map of log2 (normalized counts per million) of the top 20 DEG in W8 (red) compared to W15 (green) and W19 (blue) Sertoli cells. (*G*) IF staining of Sertoli Cells (SOX9, Cyan) confirms MYH7 (magenta) is enriched in W8 Sertoli cells but repressed with at W15 and W19. (*H*) IF staining for ESPN (magenta) a gene enriched with Sertoli cell (SOX9, cyan) maturation is expressed at W19 (arrows), not W8 or W15. Nuclei were detected with DAPI (gray). (Scale bar, 20 µm.) Statistics for (*C*) and (*F*) were FDR adjusted *P*-value < 0.05, log2FC ≥ 1.

A consistent hallmark of Sertoli cells across developmental time is that they express the transcription factor SOX9 in the nucleus, and the hormone AMH in the cytoplasm. Interestingly, we observed heterogeneous AMH protein expression in SOX9-positive cells (white arrows) at W19 (Fig. 2*A*). Therefore, to identify Sertoli cells in the 10X single cell RNA-Seq dataset over time, we reclustered the testicular samples that did not go through an enrichment protocol, that way all Sertoli cells were processed using the same single cell dissociation approach (N = 10) (*SI Appendix*, Fig. S3 *A* and *B*). Cell identity was assigned to the clusters using diagnostic markers according to Fig. 1. This reclustering resulted in 6 major cell types (*SI Appendix*, Fig. S3 *A* and *B*). The Sertoli cells in this reclustering also demonstrated distinct transcriptomic features when comparing Sertoli cells at W8 relative to later time points (W15 and W19) (*SI Appendix*, Fig. S3 *A* and *B*). Therefore, we moved forward with analysis to identify differentially expressed genes (DEGs) between Sertoli cells at the three time points.

To achieve this, we first performed filtering and normalization using summarized raw counts from the 10 samples. Box plots using the Trimmed mean of M values (TMM) method (*SI Appendix*, Fig. S3*C*) and density plots of normalized log-CPM values (*SI Appendix*, Fig. S3*D*) reveal that after filtering and normalization, the samples show similar global expression values and therefore identification of DEGs is likely to be biologically meaningful (*SI Appendix*, Fig. S3 *C* and *D*). Using an unsupervised multidimensional scaling (MDS) plot, major variations between the 10 samples could be identified in dimension 1. Specifically, in dimension 1, the transcriptional signature of W8 Sertoli cells separates from the W15 and W19 Sertoli cells (Fig. 2*B*, black dotted lines). To identify DEGs of 2-fold or greater between the groups, the limma package with empirical Bayes moderation was used (23) with a false discovery rate (FDR) adjustment of *p*<0.05 for statistical significance (Dataset S1A). This analysis identified around 1,000 statistically significant DEGs when comparing W8 to W15 and W8 to W19 Sertoli cells, with no statistically significant DEGs meeting thresholds when comparing W15 to W19 (Fig. 2*C*). Among the DEGs, 323 up-regulated genes and 342 down-regulated genes overlap in two comparisons (Dataset S1B). In addition, we also performed subclustering for the Sertoli cell populations, showing two major subclusters which are correlated with developmental age (*SI Appendix*, Fig. S4 *A* and *B* and Dataset S1C). These data suggest that a major transcriptional shift in Sertoli cell identity occurs between W8 and W15 of prenatal life, but that limited transcriptional changes occur between W15 and W19.

To identify whether specific functional terms were enriched in Sertoli cells at W8 compared to W15 and W19, we performed Gene Ontology (GO) analysis on DEGs (Dataset S1A). Given that the W15 and W19 groups show no statistically significant genes, we compared the n = 4 W8 samples to the n = 6 “later” samples composed of W15 and W19 Sertoli cells (Fig. 2 *D* and *E*). Gene ontology analysis of the DEGs upregulated at W8 compared to the later time points included terms such as “Role of JAK2 in Hormone-like Cytokine Signaling” (Fig. 2*D*). In contrast, GO terms that were enriched in the later W15/W19 included Sertoli cell terms such as “Sertoli cell-Sertoli cell junction signaling” and “Germ Cell Sertoli Cell Junction signaling” which collectively imply that the Sertoli cell identity matures even while Sertoli cell identity genes such as SOX9 remain consistent. In addition, coincident with Sertoli cell maturation at W15/W19, additional GO terms associated with upregulated genes in the later time points include those associated with FGF signaling, retinoic acid response, and actin cytoskeleton. However, Sertoli cells in these stages have not yet reached sufficient maturation to express AR and FSHR (*SI Appendix*, Fig. S4 *C* and *D*).

A heat map was created to display normalized relative expression levels of statistically significant DEGs in the 10 biological samples (Fig. 2*F*). Genes such as Aldehyde dehydrogenase 1 A3 (*ALDH1A3*) are enriched in W15/W19 Sertoli cells, as well as *ESPN,* a protein involved in actin bundling that has previously been described as an essential protein for hearing (24). Genes that show increased expression at W8 relative to later time points include *MYH7*, a protein found in muscle fibers and is associated with hypertrophic cardiomyopathy (25). To determine whether changes can be detected at the protein level, we stained the rhesus testis at W8, W15, and W19 for MYH7 (Fig. 2*G*) and ESPN (Fig. 2*H*). These data show that MYH7 protein is enriched in the SOX9+ Sertoli cells at W8 as predicted from the scRNA-Seq. Whereas ESPN protein is enriched in the cytoplasm of Sertoli cells at W19 and not at W8 and W15. Adult mouse testis was used as a positive control for staining where ESPN forms ectoplasmic specializations between neighboring Sertoli cells (*SI Appendix*, Fig. S3*E*) (26). Curiously, we did not identify ESPN at W15 which suggests that protein expression is regulated differently than the RNA. As ESPN is expressed only in Sertoli cells at W15 and W19, not in interstitial cells (*SI Appendix*, Fig. S3*D*), the positivity in the interstitium in Fig. 2*H* could be autofluorescence of the mouse host secondary antibody against blood cells in human fetal tissue. To further characterize Sertoli cell protein expression, we also evaluated GATA4, a critical transcription factor for Sertoli cell specification, and found that it was homogeneously expressed in SOX9-positive cells during fetal development (*SI Appendix*, Fig. S4*G*). Taken together, our data demonstrate that Sertoli cells transcriptionally mature with developmental time, and we identify new genes/proteins that distinguish Sertoli cells over time.

### The Testicular Interstitium Undergoes a Major Transcription Shift Between W8 and W15.

**Fig. 3.**
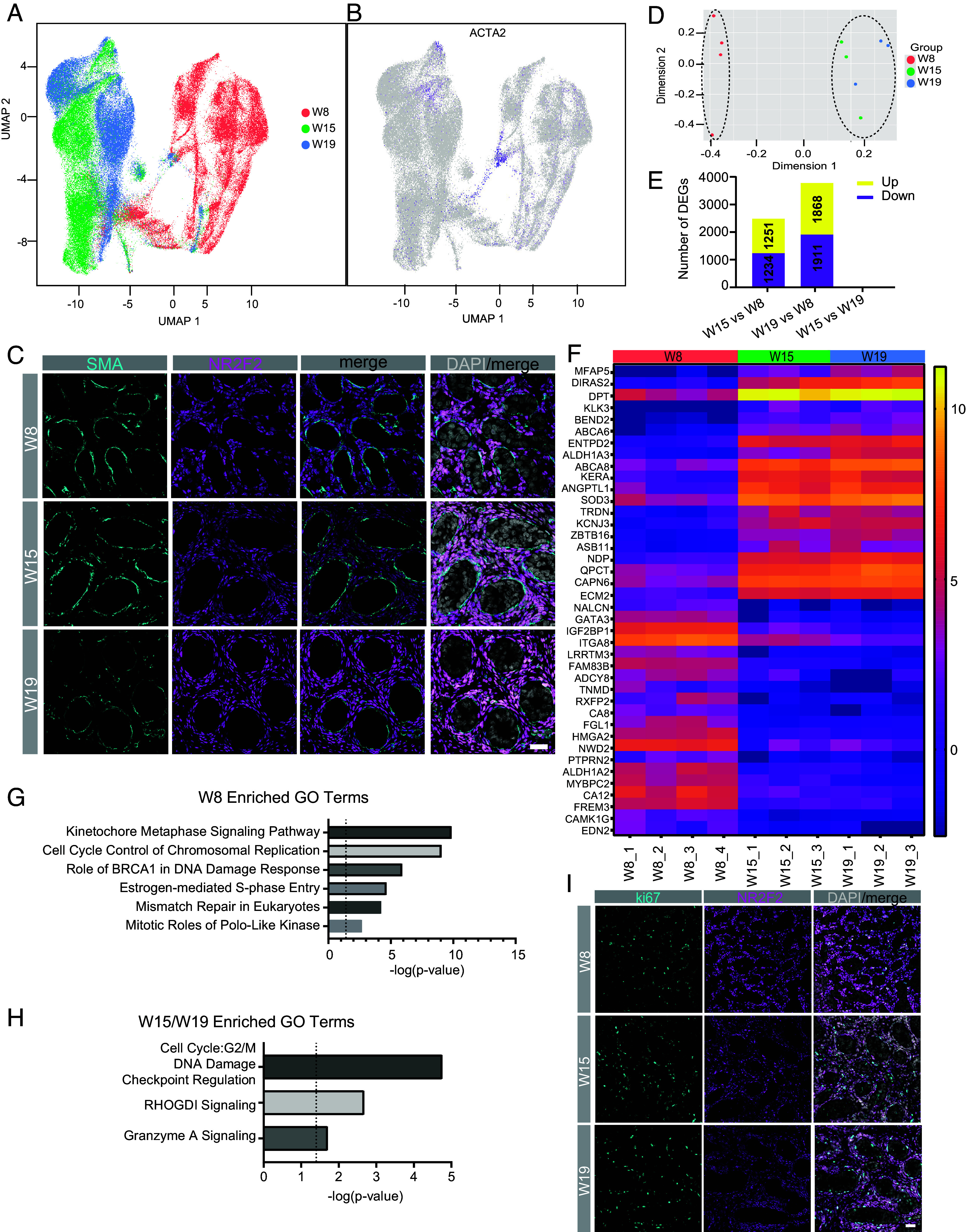
The interstitial cell identity matures between the first and second trimesters. (*A*) UMAP plot of NR2F2+ TCF21+ interstitial cells from *SI Appendix*, Fig. S4*A* (W8 = red, N = 4; W15 = green, N = 3; W19 = blue, N = 3). 198,449 interstitial cells analyzed. (*B*) UMAP features plot of (*A*) displaying ACTA2 [putative peritubular myoid cells (PMCs)]. (*C*) IF staining for the ACTA2 protein product, SMA (cyan) identifies SMA+ NR2F2+ cells surrounding the tubules. (*D*) Unsupervised MDS plot of interstitial cells from biological replicates at W8, W15, and W19. (*E*) Bar graph showing the total number of DEGs in the pair-wise comparisons shown. The number of DEGs is shown on the bar. (*F*) Heat Map of log2 (normalized counts per million) of the top 20 up-regulated and top 20 down-regulated DEGs at W8 (red), W15 (green), & W19 (blue). (*G* and *H*) IPA analysis of pathways enriched in the interstitial cells of first trimester testes relative to the second and third trimesters (*G*) and pathways enriched as interstitial cells mature in the second and third trimesters (*H*). (*I*) IF for MKi-67 (cyan) is primarily found localized to the interstitium, and not Sertoli cells (SOX9, magenta). Quantification of the IF is shown in *SI Appendix*, Fig. S5*G*. (Scale bar, 20 µm.) DAPI (gray) detects nuclei. For (*E*) and (*H*), statistics were calculated using an FDR adjusted *P*-value < 0.05, log2FC ≥ 1.

Given that Sertoli cells demonstrate a major transcriptional shift between W8 and W15, we next hypothesized that cells in the interstitium are also likely undergoing similar transcriptional maturation given that the Sertoli cells are embedded in the interstitial niche. Given that interstitial fibroblast cells show cellular differences between W8 and W15/W19 (Fig. 1 *D* and *E* and *SI Appendix*, Fig. S3 *A* and *B*), we proposed that the transcriptional identity of testicular interstitial fibroblasts may also be changing over developmental time. To address this, we reanalyzed the scRNA-Seq specifically the interstitial fibroblasts from n = 10 testis samples from *SI Appendix*, Fig. S3*B*. Prior to reanalysis, we performed UMAP representation of the interstitial cells for the age, and as marked by *NR2F2* and *TCF21* gene expression (*SI Appendix*, Fig. S5*A*). This analysis reveals a clear separation between W8 and W15 interstitial fibroblasts with small cluster of cells at W8 overlapping with interstitial fibroblasts at W15 and W19 (Fig. 3*A*). Screening for genes that are enriched in this cluster identified *ACTA2* (Fig. 3*B*), a gene that encodes for smooth muscle actin (SMA) protein a marker of peritubular myoid cells (PMCs) (27). PMCs surround the testes in humans and are first observed at 10 wpc (28). To examine SMA expression during rhesus testis development, we performed immunofluorescence for SMA together with the interstitial protein marker NR2F2 (Fig. 3*C*). This result shows that as early as W8 (equivalent to 7 to 8 wpc in humans), SMA protein can be detected in a subpopulation of NR2F2+ cells in the testicular interstitium surrounding the seminiferous cords.

To identify additional changes in gene expression between W8 and W15 we again performed filtering and normalization using summarized raw counts of the interstitial cells (*SI Appendix*, Fig. S5 *B*, *C*) followed by pseudo bulk analysis of the interstitial cells (Dataset S2A). Using an unsupervised MDA plot of the pseudo bulk RNA-seq results, our data reveal that interstitial fibroblasts at W8 are transcriptionally distinct in dimension 1 compared to interstitial fibroblasts at W15 and W19 (later time points) (Fig. 3*D*). Identifying statistically significant DEGs between W8 and W15 or W19 identifies thousands of differentially expressed genes between W8 and W15 and W8 and W19 (Fig. 3*E*), with no statistically significant genes that met our thresholds between W15 and W19. This is a similar scenario to what was observed for Sertoli cells.

To demonstrate relative expression differences of the top 40 DEGs in the ten samples we created a heatmap (Fig. 3*F*). Notably transcription factors like *GATA3* are expressed at statistically higher levels in the interstitial cells at W8 compared to later stages (Dataset S2A), with *GATA3* previously identified as a marker of immature testicular progenitor cells (29). The RNA binding protein IGF2BP1 was also highly expressed in W8, which is known to be required for spermatogenesis in mice (30). Microfibril-Associated Glycoprotein 5 (*MFAP5*) has not been described in testis cells but has been identified in cancer associated fibroblasts where it is associated with extracellular matrix remodeling (31). DIRAS2 has been demonstrated to promote renal cell carcinoma formation by activating the mitogen-activated protein kinase pathway (32). Further investigation is necessary to ascertain the role of this pathway in testicular development. To evaluate biological pathways that are changing in the interstitial fibroblasts between W8 and the later time points, we performed Gene Ontology (GO) analysis and discovered genes associated with mitosis and DNA damage response, suggestive of extensive interstitial cell proliferation at W8 (Fig. 3*G*). In contrast, DEGs that are upregulated at the later time points indicate G2/M checkpoint regulation (Fig. 3*H*).

Given the abundance of cell-cycle regulation genes that emerged in the G0 analysis, we displayed some of these genes on the interstitial cell UMAP. *MKi67* is a gene that denotes cycling cells are enriched in a subset of W8 cells. Similarly, *PCNA, AURKA, CCNA2, CCNB1,* and *TOPA2A* are all enriched at W8 relative to W15 and W19 interstitial cells (*SI Appendix*, Fig. S5*D**).* In the subclustering of interstitial cells, clusters were also defined by the proliferative index, as indicated by gene expression, and proliferative interstitial cells are more common in cluster 3 and W8 samples (*SI Appendix*, Fig. S5 *E* and *F* and Dataset S2B). To evaluate the proliferation of interstitial cells, we stained samples at W8, W15, and W19 and identified Ki67 cells at W8 with lower levels at W19 (Fig. 3*I*). Quantification of Ki67-positive in NR2F2+ cells also showed that interstitial cells were highly proliferative at W8 (11.55%) compared to W15 (6.18%) and W19 (5.94%), while proliferative NR2F2- cells gradually increased (2.79% at W8, 14.42% at W15, and 25.7% at W19) (*SI Appendix*, Fig. S5*G*). Taken together, interstitial fibroblast cells undergo a major transcriptional shift between W8 and W15 with no major transcriptional changes between W15 and W19.

Finally, as the fetal Leydig cells are embedded in the interstitial niche, we also interrogated this cell population over time by extracting all cells from *SI Appendix*, Fig. S3*B* defined as fetal Leydig cells and displaying the data on a new UMAP. Subclustering of fetal Leydig cells showed two subpopulations. Cluster 2 appeared specifically at W8 (*SI Appendix*, Fig. S6*A*), as expected in *SI Appendix*, Fig. S3*B*, where specific markers are mostly involved in metabolic pathways and enzymes such as “fatty acid beta-oxidation using acyl-CoA dehydrogenase” (Dataset S3). In fetal stages, key enzymes for testosterone production in addition to *CYP17A1*, such as *STAR*, *CYP11A1*, were highly expressed, but later markers including *HSD3B1* and *HSD17B1* did not yet show expression in fetal Leydig cells (*SI Appendix*, Fig. S6 *B* and *C*). Therefore, similar to interstitial fibroblasts, the fetal Leydig cells also undergo transcriptional maturation between W8 and W15.

### MSL3 Is Expressed in Fetal Germ Cells During the PGC to Fetal Spermatogonia Transition.

**Fig. 4.**
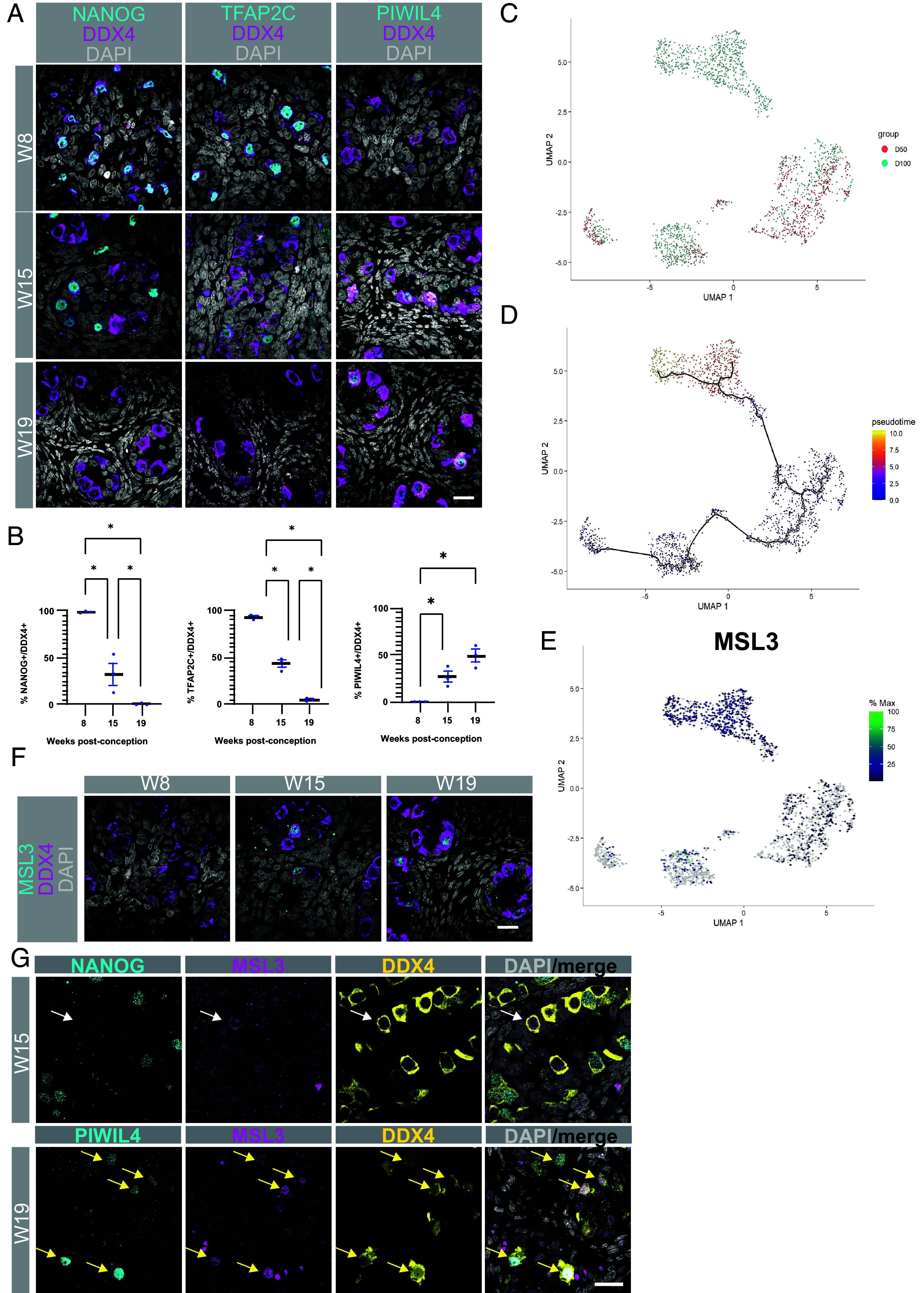
MSL3 marks the male rhesus macaque second trimester germline. (*A*) *Left*, IF for NANOG (cyan) and DDX4 (magenta); Center, IF for TFAP2C (cyan) and DDX4 (magenta); *Right*, IF for, PIWIL4 (cyan), DDX4 (magenta). (*B*) Quantification of the % DDX4 positive germ cells for proteins detected in (*A*). Quantification was performed in three sections, each 50 µm apart, under blinded conditions. (*C*) UMAP of the Germ Cell cluster defined in Fig. 1*E* (1,967 number of cells) showing a distinct cluster of W15 germ cells. (*D*) Pseudo time trajectory analysis on Germ cells. (*E*) UMAP plot of a diagnostic gene that changes with pseudotime, MSL3. (*F*) IF for DDX4 (magenta) with MSL3 (cyan). (*G*) IF for DDX4 (yellow) with NANOG/PIWIL4 (cyan) and MSL3 (magenta). Scale bar, 20 µm and nuclei are stained with DAPI (gray).

Prior analysis of human testis development revealed changes in germline identity with progression toward PIWIL4+ spermatogonia between 12 to 17 wpc (11). To address this in rhesus macaque development, we performed immunofluorescence for DDX4, a pan–germ cell marker, together with NANOG and TFAP2C, two markers of PGCs, as well as PIWIL4, a marker of fetal spermatogonia (Fig. 4 *A* and *D*). At W8, almost 100% of DDX4+ germ cells are positive for NANOG and TFAP2C, indicating that germ cells at W8 are PGCs (Fig. 4*A*). This is consistent with what was previously described at Carnegie Stage 23 in the rhesus macaque (33). At W15, the identity of germ cells is changing, with around 50% or fewer germ cells positive for NANOG or TFAP2C (Fig. 4*B*). Evaluation of PIWIL4 reveals the initiation of protein expression between W8 and W15 and 26.53% of DDX4+ germ cells positive for PIWIL4 at W15 (Fig. 4*B*). By W19 48.67% of germ cells had progressed to a PIWIL4 identity.

To further interrogate the germ cell identity, we extracted the DDX4+ germ cell cluster from Fig. 2 and displayed the germ cells on a new UMAP (Fig. 4*C*). This identified four major clusters of germ cells with one cluster enriched in germ cells from W15 suggesting that the germ cells can be aligned computationally using pseudotime. To achieve this, we performed Monocle lineage trajectory on the germ cells which predicted a lineage trajectory from W8 to the germ cells enriched in the W15 cluster (Fig. 4*D*). Through this analysis, we identified a germ cell-expressed gene in the testis, male sex-lethal 3 (*MSL3*), which is uniquely enriched in the germ cells at W15 (Fig. 4*E*). MSL3 protein is found in spermatogonial cells in the adult human and rhesus testis. To evaluate whether MSL3 protein can be identified in the fetal testis, we stained for MSL3 at W8, W15, and W19 and show that, as predicted, MSL3 protein is first detected at W15 and continues to be expressed in fetal germ cells at W19 (Fig. 4*F*). At W15, MSL3/DDX4 double positive germ cells were negative for NANOG in early germ cells at W15 (white arrow), while at W19 MSL3/DDX4 double positive germ cells were also positive for PIWIL4 (yellow arrows) (Fig. 4*G*). Taken together, RNA-Seq analysis of germ cells demonstrates that as the Sertoli and interstitial niches are changing between W8 and W15, the fetal germline initiates sex-specific differentiation into fetal stage spermatogonia expressing PIWIL4 and MSL3.

### Sex-Determination Occurs Between W5 and W6 of Rhesus Macaque Development.

**Fig. 5.**
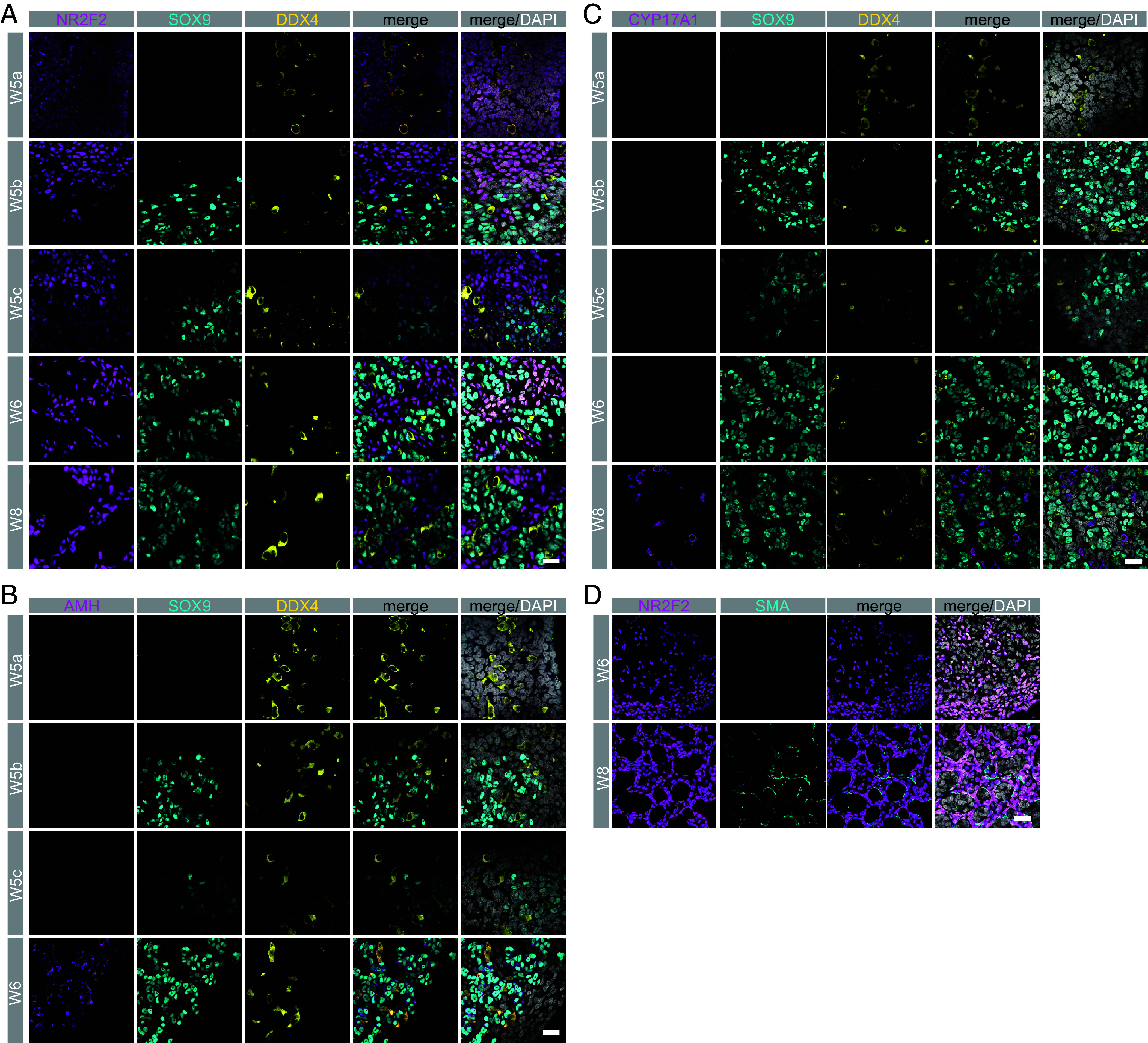
The rhesus testis originates from NR2F2+ progenitors. (*A*) IF staining for SOX9 (cyan) and NR2F2 (magenta) in W5 (N = 3), W6 (N = 1), and W8 (N = 5). Starting at W5, SOX9+, NR2F2 negative cells are identified in 2/3 samples. (*B*) AMH (magenta) expression is initiated by W6 in SOX9+ Sertoli cells. (*C*) CYP17A1 (magenta) a marker of fetal Leydig cells is first observed at W8 in the interstitial space. DDX4 (yellow) marks germ cells (*A*–*C*). (*D*) SMA (cyan) a marker of PMCs is first identified at W8 in the interstitium. For *A*–*D* DAPI (gray) stains nuclei and scale bar, 20 µm.

Given that Sertoli cell, interstitial fibroblast, PGC, Leydig, and PMC identity can each be identified in the testis of the rhesus embryo at W8, we took advantage of the time-mated breeding program to determine when these lineages emerge in the rhesus, with a focus on W5-W6 (corresponding to CS12-CS21 of embryo development) with W8 used as a positive control. Biological sex for samples at W5-W6 was performed by PCR given that external sex characteristics were not visible. At W5, the genital ridge expands such that the NR2F2+ interstitial cells are now enriched at the connection between nascent gonad and mesonephros (*SI Appendix*, Fig. S7*A*) and laminin deposition can be observed in the nascent gonad (*SI Appendix*, Fig. S7*B*). At W6, the testis is a simple organ with Laminin+ basement membranes separating putative cords from the NR2F2+ interstitial cells (*SI Appendix*, Fig. S7 *A* and *B*). Evaluation of SOX9, one of the earliest proteins to be expressed in Sertoli cells after SRY, revealed heterogenous expression at W5 (Fig. 5*A*). Specifically, 2/3 samples at W5 expressed SOX9+ cells in the vicinity of DDX4+ germ cells. For the sample that did not express SOX9 (W5a), NR2F2 expression was still detected in the nascent gonad (Fig. 5*A* and *SI Appendix*, Fig. S7*B*). Taken together, this suggests that sex determination and specification of Sertoli cells is initiated at W5 of rhesus testis development.

AMH, an alternate marker of Sertoli cells, was analyzed in the three samples at W5 as well as W6. These data show that AMH is not expressed in any sample at W5 and instead is expressed by Sertoli cells at W6 and continues to be expressed in SOX9+ Sertoli cells at W8. Therefore, AMH expression is initiated after SOX9 in the Sertoli cells of the embryonic testis (Fig. 5*B*). In addition to AMH, another hormone produced by the fetal testis is testosterone. Testosterone is produced by the Leydig cells, with the final step in testosterone production involving the enzyme CYP17A1. Therefore, CYP17A1 expression is a useful indicator of Leydig cell identity as well as the capacity for the embryonic testis to convert cholesterol to testosterone. Our data shows that CYP17A1 is not detected until W8 where it is found in the interstitium of the W8 testis (Fig. 5*C*). In our 10X Genomics dataset, we identified *ACTA2* PMCs at W8, which we verified using immunofluorescence for SMA (Fig. 3*B*). Using the embryonic samples, we stained for SMA at W5, W6, and W8 and found that SMA protein expression is first initiated in a subpopulation of cells at W8 in the interstitium and is not detected at earlier time points. A model for rhesus macaque testis development is shown in (*SI Appendix*, Fig. S7*C*).

## Discussion

The third trimester of pregnancy represents a critical period of fetal development, marked by rapid growth and significant physiological maturation in humans. This final stage of gestation, which typically spans from W28 to birth in humans and W16 to birth in rhesus macaque, is characterized by substantial increases in fetal size and weight, alongside crucial developments in organ systems and neurological functions (34, 35). The third trimester is particularly important for the differentiation of the reproductive system and the establishment of endocrine functions that will be essential throughout postnatal life. In male fetuses, testosterone levels, which peaked during mid-gestation, begin to decline, while estradiol levels rise in female fetuses (36, 37). This hormonal milieu plays a pivotal role in the final stages of reproductive organ development and may have enduring effects on brain organization and future reproductive function (38, 39). During this stage, the testis undergoes crucial phases of differentiation and maturation, which are essential for the establishment of male reproductive function postnatally. The testis begins its descent into the scrotum, with almost complete descent by the end of this trimester in most cases (9). Seminiferous cord organization becomes more distinct, with small opening of the cords becoming clearer and more organized as fetal development progresses (10). However, limited molecular knowledge has accompanied these histological changes in the third trimester.

The recent advancements in single-cell RNA sequencing technologies have significantly enhanced our comprehension of the nascent gonadal and testicular development in humans, particularly during the first and early second trimester stages (1, 11, 15, 40). These studies have provided an unprecedented atlas of human gonadal development revealing the complex cellular dynamics and gene expression patterns occurring during sex determination and early gonadogenesis. However, the ethical and practical constraints surrounding late second and third-trimester pregnancies have resulted in a paucity of molecular information about late-stage fetal testicular development in humans. To address this knowledge gap, we employed the rhesus macaque (Macaca mulatta) as a model organism, thereby a comprehensive analysis of testis formation from the first to the third trimester.

Our single-cell RNA-seq analysis of rhesus macaque testicular development revealed several important insights. Notably, we observed that most molecular changes associated with cellular identity occurred during the first and second trimesters. This aligns with findings in human fetal development, where most cell types are specified before W12 (1, 11). In the rhesus model, we found that most cell types were already specified by W8. Interestingly, we observed a significant proliferation of interstitial cells at W8 in rhesus, which corresponds to the increase in the proportion of interstitial cell population observed between W12 to W14 in humans (1). These interstitial cells exhibited a mesenchymal morphology at W8 but progressively acquired a more fibroblast-like morphology as development progresses, suggesting a dynamic differentiation process into more mature cell types (41), along with dramatic changes in gene expression and proliferation index between the first and second trimesters. Our data indicated that pro-spermatogonia formation is still ongoing in the third trimester, as evidenced by incomplete PIWIL4 expression, which is first seen at W15. Additionally, we observed the expression of MSL3 in germ cells, which was also initiated at W15, raising questions about its function in this context. MSL3, known for its role in X chromosome dosage compensation, may play an as-yet uncharacterized role in germ cell development or epigenetic regulation during spermatogenesis (42).

Our analysis revealed an intriguing pattern of AMH protein expression in Sertoli cells during rhesus monkey fetal testis development. While most gene expression changes in Sertoli cells occurred between W8 and W15, we observed heterogeneous AMH protein expression in SOX9-positive Sertoli cells at W19. This finding contrasts with observations in mice, where AMH expression is relatively uniform throughout fetal and early postnatal testis development (43). In humans, AMH expression is reported to persist throughout all fetal stages, with high serum levels maintained until the peripubertal period (44, 45). The heterogeneous expression observed in rhesus at W19 may represent a transitional state between the fetal and postnatal periods, potentially reflecting the onset of Sertoli cell maturation. This maturation process in humans is associated with the expression of androgen receptors and testosterone sensitivity and a subsequent decrease in AMH levels around puberty (45, 46). Our findings suggest that rhesus monkeys may undergo a similar, but possibly more gradual, transition in AMH expression patterns during late fetal development to prepare for perinatal maturation of the testicular environment earlier than in humans. However, the temporal sequence of maturation of Sertoli cells involving expression of androgen receptors and sensitivity to testosterone remain to be elucidated.

Additionally, in conjunction with the potential maturation of interstitial cells, the localization of Sertoli cell nuclei at W19 shifted to the outer layout of cords, with closer association to the basement membrane than W15 where the nuclei tended to be centrally localized. Furthermore, the nascent opening within the cords became increasingly discernible, but not yet equivalent to lumen formation after puberty or in adult Seminiferous tubules as shown by previous studies in humans and Rhesus Macaque (10, 47, 48). To eliminate the possibility of artifacts by fixation, the nascent opening will require further studies to confirm the situation under which seminiferous cords exhibit nascent openings. This could be achieved using alternative fixation such as Bouin’s solution or glutaraldehyde. The transcriptional change in Sertoli cells of the seminiferous cords is closely associated with the increasing complexity of the surrounding interstitial matrix and blood vessels, contributing to the structural organization of the testis (49). Concurrently, elongated cells in the interstitial compartment provide critical support for seminiferous cords through structural maintenance and paracrine signaling, facilitating interactions between Sertoli cells and germ cells (50, 51).

In summary, this study offers a comprehensive analysis of testicular development across the three trimesters, employing the rhesus macaque as a translationally relevant primate model. Our findings indicate that most testicular-specific cell types (Sertoli, PTM, and Leydig cells) present in the fetal stage are established before W8, with an accuracy of ±1 d. For developmental events occurring within time spans of hours additional time points will be needed, possibly using in vitro fertilization. Substantial alterations in the transcriptional signatures of Sertoli and interstitial fibroblast and Leydig cells (but not PTM) occur between W8 and W15. In contrast, alterations in structural and protein expression were observed, rather than changes in gene expression at W19. Even though gene expression in the same cell types was highly similar between W15 and W19, the actual protein expression, as determined by immunostaining, exhibited differential patterns that could be observed in the fetal testis closer to birth. These studies provide valuable insights into the intricate processes involved in the formation and maturation of testicular structures, which are critical for reproductive function. Our findings will extend the temporal scope of these investigations, offering crucial insights into the molecular dynamics of testicular development during the critical but understudied window of fetal development just prior to birth. Future studies should explore the epigenetic mechanisms, proteomics, and translational control processes that regulate the maturation of testicular somatic cells during fetal and early postnatal development.

## Materials and Methods

### Time-Mated Breeding of Rhesus Macaque and Sample Processing.

Time-mated breeding of rhesus macaque adult animals was performed as previously published (33, 52). Briefly, estradiol was measured in the female animal daily from day 5 (D5) to D8 after menses began. Once estrogen levels had risen above baseline (>50 pg/mL), a known fertile male was paired with the female. Twenty-four hours after ovulation as measured by the estrogen peak (2) the male was removed. The length of time males and females were paired together was 3 to 7 d total. Based on previous studies, fertilization (D1) is estimated to occur approximately 72 h after the estrogen peak (53) (*SI Appendix*, Fig. S1*A*). Pregnancies were confirmed using ultrasound and progesterone measurements. This revealed that the timing of fertilization is not accurate if the last menstrual period was used to estimate the day of conception (*SI Appendix*, Fig. S1*B*). For embryonic time points starting at W8, biological sex was confirmed using cell-free DNA analysis (54) following a blood draw of the confirmed pregnant animal. Conceptuses were collected following C-section (n = 3 at W5, n = 1 at W6, n = 5 at W8, n = 4 at W15, n = 3 at W19). Approval by the Oregon National Primate Research Center (ONPRC) Institutional Animal Care and Use Committee (IACUC) was obtained before rhesus macaque time-mated breeding experiments were performed. UCLA approval for the use of the ONPRC animal resource was also obtained. For conceptus samples >W8, the testes were isolated and shipped in conical tubes containing Hanks’ Balanced Salt Solution (HBSS, Gibco) on ice from ONPRC overnight to UCLA. Upon receipt at UCLA, one testis was processed for histology (described below) and the other testis was processed for single cell RNA-Seq (scRNA-seq) using 10x Genomics, as described in *SI Materials and Methods*.

## Supplementary Material

Appendix 01 (PDF)

Dataset S01 (XLSX)

Dataset S02 (XLSX)

Dataset S03 (XLSX)

## Data Availability

The scRNA-seq of this article has been deposited in the Gene Expression Omnibus (GEO: GSE263989) (52).
