# Supplementary material for: Testicular somatic and germ cell maturation during rhesus macaque development
Source: Proc Natl Acad Sci U S A. 2025 Jun 26;122(26):e2419995122. doi: 10.1073/pnas.2419995122 (PMC12232671; doi:10.1073/pnas.2419995122)
Supplement: Supplementary file 1 — Appendix 01 (PDF) [file pnas.2419995122.sapp.pdf]

PNAS

Supporting Information for
Testicular Somatic and Germ Cell Maturation During Rhesus Macaque Development.

Enrique Sosa[1,2,3], Sissy E. Wamaitha[1,2,3], Fei-man Hsu[1,2,3], Mary Jasmine D. Lara[1,2,3], Kiana Oyama[4], Maggie Custer[4], Melinda Murphy[4], Jon D. Hennebold[4,5], *Young Sun Hwang*[1,2,3], Amander T. Clark[1,2,3]

Affiliations
1 UCLA Department of Molecular, Cell and Developmental Biology, UCLA, Los Angeles, USA
2 UCLA Eli and Edythe Broad Center of Regenerative Medicine and Stem Cell Research, Los Angeles, USA
3 UCLA Molecular Biology Institute, University of California Los Angeles, CA, USA
4 Division of Reproductive & Developmental Sciences, Oregon National Primate Research Center, Beaverton, USA
5 Department of Obstetrics and Gynecology, Oregon Health & Science University, Portland, USA

Corresponding Author: Amander T. Clark

Co-corresponding Author: Young Sun Hwang

Email: clarka@ucla.edu

This PDF file includes:

SI Materials and Methods
Figures S1 to S7
Tables S1 to S4
Legends for Datasets S1 to S3
SI References

Other supporting materials for this manuscript include the following:

Datasets S1 to S3

SI Materials and Methods

Genotyping biological sex

For embryonic time points before day 50, biological sex was confirmed using polymerase chain reaction (PCR) on genomic DNA isolated from conceptus tissue after the conceptus was collected. Briefly, the Y-chromosome specific genes DYS14 and SRY were PCR amplified from rhesus macaque genomic DNA (gDNA), purified, cloned into a pGEM-T Vector, and transformed into JM109 competent cells (pGEM-T Easy Vector Systems, Promega, Madison, WI). Conventional PCR was performed to identify clones with the correct insert, and subsequent plasmid DNA was extracted and purified using the GeneJET Plasmid Miniprep Kit (Thermo Fisher Scientific, Waltham, MA). Standard curves were generated using purified plasmid DNA and used to determine DYS14 and SRY levels. To perform fetal sex determination, maternal blood was collected from pregnant rhesus macaques at W8 – W19 to obtain circulating cell-free DNA (ccfDNA). Blood samples (4 ml collected into a no additive vacutainer tube) were centrifuged, and the resultant serum was immediately used to isolate ccfDNA using the QIAmp MinElute ccfDNA Kit (Qiagen, Germantown, MD) following the manufacturer's instructions. To determine sex from fetuses <W8, fetal tissue was collected at the time of necropsy and a small piece of tissue was used for gDNA isolation using the GeneJET Genomic DNA Purification Kit (Thermo Fisher Scientific, Waltham, MA) according to the manufacturer's instructions. DYS14 and SRY presence were determined by qPCR in the extracted ccfDNA and gDNA samples. Fetal sex was determined to be female if DYS14 and SRY genes were not detected, whereas the sex was determined to be male if DYS14 and SRY genes were detected and at a level that was above the minimum plasmid standard.

Fetal Gonad Dissociation

Two different approaches were used for tissue dissociation. The first approach is called "All" cells. In this approach, testicular samples were washed in Dulbecco's Phosphate Buffered Saline (DPBS, Gibco 14190-144) before removing any non-gonadal tissues, such as adipose or connective tissues. Testes were then washed two times in DPBS and digested in Dissociation Medium consisting of Collagenase IV (1g/ml, Gibco 17104019), 10% Fetal Bovine Serum (FBS, Hyclone SH30070.03), Dispase II (10mg/ml; Gibco 17105041), DNAse I (2.5mg/ml; Worthignton LS002147) in DPBS. Digestion of testicular tissues was carried out by incubating the tissue at 37°C for 8-15 minutes, with pipetting at every 5-minute interval to mechanically break down the tissues into a single cell suspension. Refer to Supplemental Table 1 for the testis samples used in the "All" approach. The second approach for tissue dissociation, termed "Tubule Enriched," was used to enrich for cells within the tubules (Sertoli cells and germ cells). In this approach, testis weights, without the Tunica Albiguenia, were taken and for every 100mg of testis weight 1ml of Dissociation buffer #1 was prepared containing Collagenase IV (0.5mg/ml; Worthington-LS004188) diluted in Hanks Balanced Salt Solution (HBSS; CEllgro-21-020-CV). Dissociation buffer #2, consisted of DNAse I (2.5mg/ml; Worthignton LS002147) and 0.25% Trypsin (Gibco-BRL, 25200-056). Prior to the addition of Dissociation media #1, the Tunica Albuginea was removed from the testis. The 1st dissociation was performed in a 37°C shaker (250rpm) for 5-8 minutes. Samples where then spun down (1600rpm) for 5 minutes, and the supernatant was removed. Next, Dissociation media #2 was added to the seminiferous tubules for 5 minutes at 37°C. Please refer to Supplemental table 1 for the testis samples used in the Tubule Enriched approach. After digestion, the cell suspension was washed with alpha-MEM supplemented with 10% FBS (Hyclone SH30070.03) by centrifuge at 1600rpm at room temperature. The cell pellet was resuspended in Fluorescence-Activated Cell Sorting (FACS) buffer then strained through a 70-micron cell strainer. The cell number was determined, and samples were sent to the UCLA Technology Center for Genomics and Bioinformatics (TCGB) for processing at a concentration range of 1,000-1,200cells/ul. All samples, except ONPRC05, were provided fresh to the UCLA TCGB sequencing core. Sample ONPRC05 was frozen as a single cell suspension due to campus closure on account of the pandemic. Once core operations resumed the sample was thawed and submitted for library preparation.

Histology

For embryo samples < W8, the embryo was removed from the extraembryonic membranes and fixed using 4% PFA for 1 hour at room temperature. Following fixation, tissue was dehydrated in a series of ethanol solutions (70, 80, 95, and 100%) and xylenes for paraffin embedding for transverse sectioning. The embryo was serially sectioned at 5 μm from tail to trunk with every 10th section stained with H&E in order to identify the location of the developing gonads. For contralateral testes > W8, the testis was fixed in 4% paraformaldehyde (Fisher Scientific) and subsequently dehydrated in a series of ethanol solutions (70, 80, 95, and 100%) and xylenes for paraffin embedding. Tissues were sectioned at 5 microns by the UCLA Translational Pathology Core Laboratory in preparation for immunofluorescence staining.

Single-cell RNA sequencing (scRNA-seq)

Libraries were prepared from the single cell suspension of rhesus macaque using Chromium Single Cell 3' v2 Reagent Kits (10X Genomics). Dissociated cells were mixed in Single Cell Master Mix generating gel beads-in-emulsions (GEMs). The RNAs in each GEM were reverse-transcribed and the resulting cDNAs were amplified by PCR. Illumina sequencer adapted sequencing libraries were constructed from the amplified cDNAs. Single-cell RNA sequencing libraries were sequenced on a Novaseq S4 flowcell, custom PE100.

Immunofluorescence staining

All immunofluorescence staining experiments were performed on paraffin embedded tissues that were first run through deparaffinization (xylenes) and ethanol steps. Slides were rehydrated and then underwent an antigen retrieval step, using a Tris-EDTA buffer (pH 9.0). Tissues were permeabilized in a 0.05% Trition-X100 solution then washed using 0.5% Tween (PBST). Tissues were then blocked with Normal Donkey Serum (Jackson ImmunoResearch) for 30 minutes at room temperature (RT) and washed with PBST. Primary antibodies were incubated overnight at 4°C. Slides were washed in PBST and then secondary antibodies were incubated at Room Temperature (RT) for 1 hour. Slides were washed and incubated with DAPI, then washed for a final time in PBST. Lastly, tissues were mounted using Prolong Gold antifade reagent (Invitrogen) and slides were sealed and allowed to cure overnight in the dark at RT.

Microscopy

Confocal images were taken using a Laser Scanning Microscope (LSM) 880 (Carl Zeiss). The objective used include a Plan-Apochromat 20×/0.8 NA, Plan-Apochromat 40×/1.4 NA M27, a Plan-Apochromat 63×/1.4 NA M27 oil immersion objective at room temperature. Acquired images were processed using IMARIS 8.1 (Bitplane). H&E slides were examined on an Olympus BX-61 light microscope using a UPlanSApo 4x/0.16 objective and a UPlanFLN 40x/1.30 oil objective. Images were acquired using the Olympus DP2-BSW software.

scRNA-seq DE

Single-cell expression matrices generated with Cell Ranger (10x Genomics) using cellular barcodes and unique molecular identifiers (UMIs) were used for single-cell (sc) transcriptome analysis. Quality assessment and quality filtering of UMIs and individual cells was performed using the scatter R package (1) with experiment specific thresholds. Data set merging, normalization, clustering, and cluster marker identification were performed using the Seurat 4.0 R package (2) with default settings. Clusters were identified as different cell types based on known markers. Pseudo-bulk differential expression (DE) analysis was performed for Sertoli Cells and Interstitial Cells. More specially, counts were summed from all cells in each cell type for each biological replicate, then the DE analysis between biological conditions for each cell type were performed using bulk RNA-seq DE methods where derived per sample counts were normalized

using the trimmed mean of M-values method (TMM) (3) and transformed to log-counts per million with associated observational precision weights using voom method (4). Gene-wise linear models comparing between developmental time points were employed using limma with empirical Bayes moderation (5) and false discovery rate (FDR) adjustment (6). For germ cells, Monocle3 was used to construct trajectories, order cells in pseudo-time, and identify genes that change with pseudo-time (7).

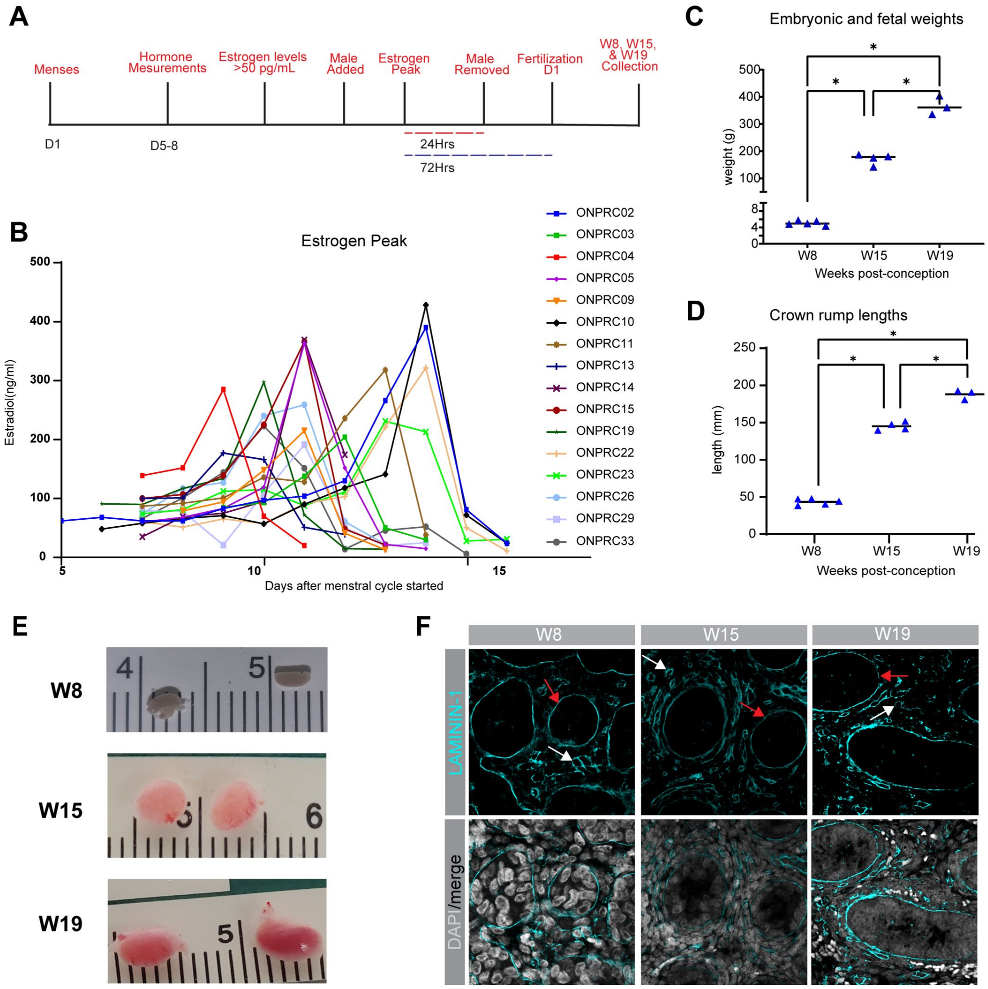

Fig. S1. Rhesus macaque embryonic and fetal testes. (A) Strategy for obtaining time-mated rhesus macaque embryonic and fetal samples using estrogen hormone measurements of the reproductive age female and precise pairing with an adult male. **(B)** Graph of estradiol peaks for each adult female used for time mated breeding in this study (N=16). **(C)** Graph of crown rump lengths, **(D)** Graph of embryo weights by the weeks (W) post-conception. **(E)** Representative photographs of testicles isolated from rhesus macaque embryos at Week (W) 50 post-conception also called Carnegie stage (CS) 23, as well as testicles from fetuses at W15 and W19. **(F)** Immunofluorescence (IF) staining for LAMININ-1 (cyan) to evaluate presence of basement membranes around the cords/tubules (red arrows). White arrows indicate LAMININ-1 deposition in the interstitium. Samples for this figure include W8, N=5 (first trimester); W15, N=4 (second trimester); W19, N=3 (third trimester). Where shown, statistical differences were calculated using One-way ANOVA (significance was accepted at p≤0.05).

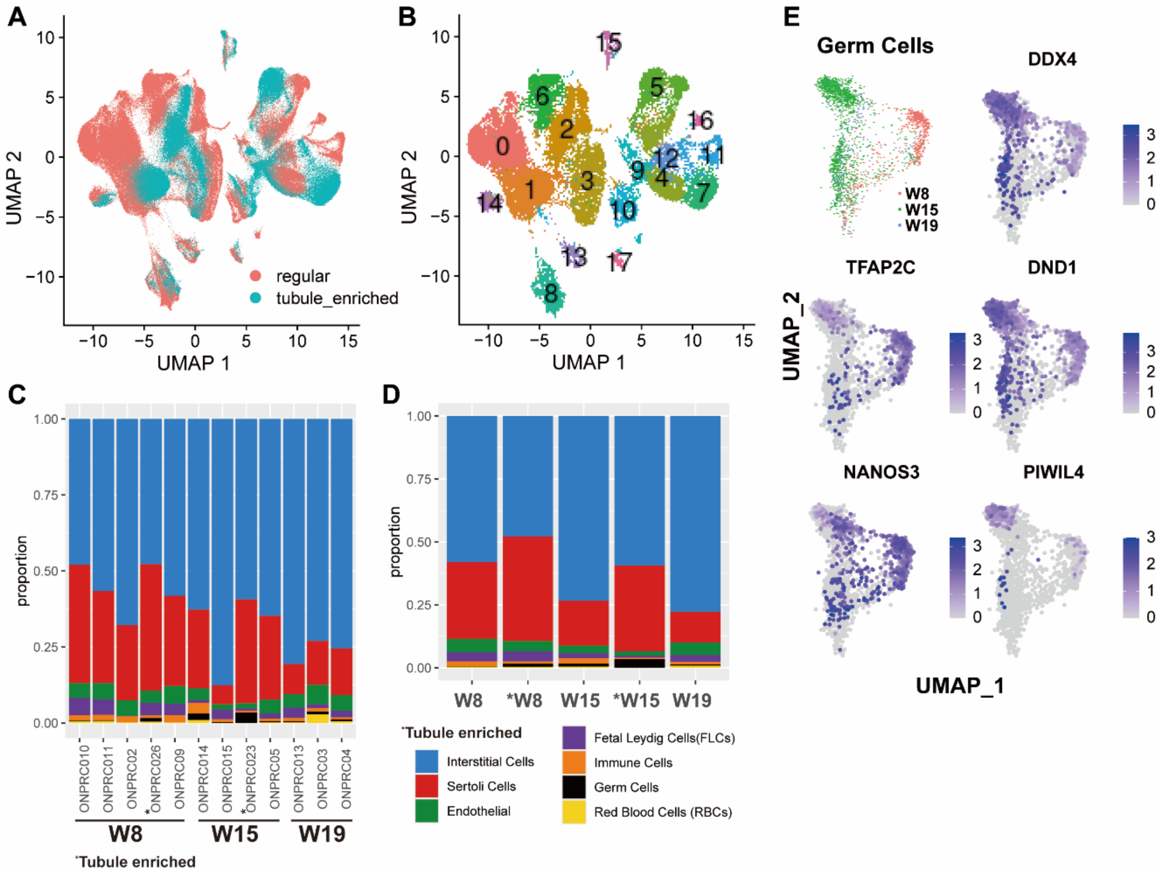

Fig. S2. Comparison of 10X Genomics data obtained from testicles with and without tubule enrichment. (A) UMAP of embryonic and fetal testes samples displayed by their respective sample processing method (n=12 samples). **(B)** UMAP Clustering analysis of samples in a revealing a total of 18 cell clusters **(C)** Bar graph comparing the different proportions of seven major cell types originating from the 18 distinct clusters in each biological sample. (W8, N=5 with N=1 tubule enriched; W15, N=3 with N=1 tubule enriched; W19, N=3). *Indicates tubule enriched sample. **(D)** Bar graph of the different cellular proportions comparing samples that underwent tubule enrichment*. Tubule enrichment led to an increase in the fraction of Sertoli cells and germ cells in the samples. **(E)** UMAP plots of germ cells annotated in Fig. 1E for the age (W8=red, W15=green and W19=blue) and germ cell marker expression; *DDX4*, *TFAP2C*, *DND1*, *NANOS3* and *PIWIL4*.

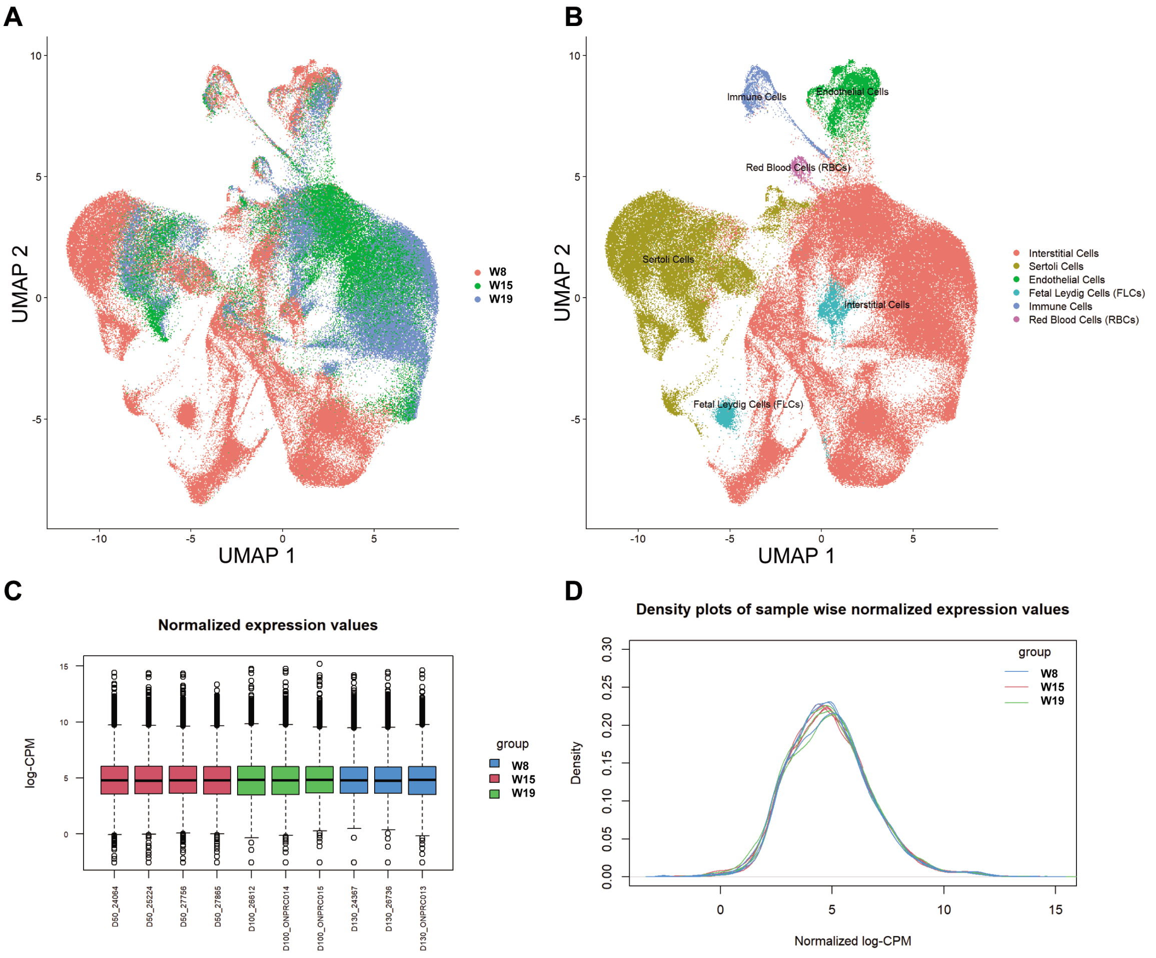

Fig. S3. 10X Genomics scRNA-seq analysis of testis somatic cells without enrichment. **(A)** UMAP of embryonic and fetal testicular cells displayed by group (W8; N=4, W15; N=3, W19; N=3). **(B)** UMAP of six major testicular cell populations from cells in (**A)** displayed with their corresponding color and label. **(C)** Summarized raw counts normalized using the trimmed mean of M values method (TMM) for genes expressed in the Sertoli cell population. Results were displayed as a box plot of normalized expression values (log-CPM). **(D)** Density plot of normalized expression values by group (Normalized log-CPM).

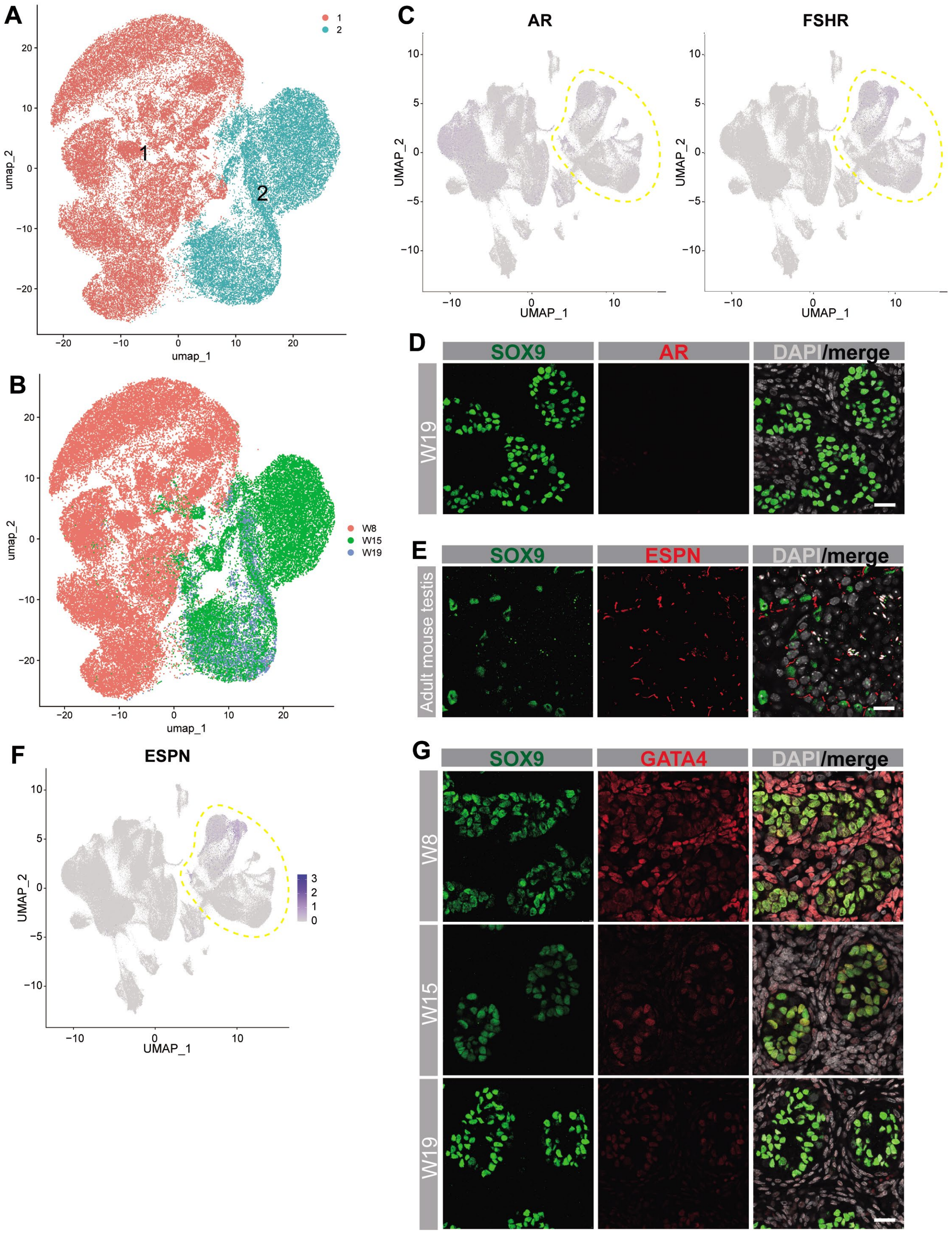

Fig. S4. Sub-clustering and maturation of Sertoli Cells. (A, B) UMAP plots for sub-clustering of Sertoli Cells showing two distinct sub-clusters **(A)** each correlating with W8 or W15/W19 **(B)**, respectively. **(C)** UMAP feature plots of testicular cells showing *androgen receptor* (*AR*, left) and *FSHR* (right) gene expression. **(D)** IF for SOX9 (green) with AR (red) at W19. **(E)** IF for SOX9 (green) and ESPN (red) on adult mouse testis. **(F)** UMAP feature plot for ESPN gene expression in testicular cells. (G) IF for SOX9 (green) and GATA4 (red) at W8, W15 and W19. The yellow

dotted circle represents Sertoli Cell population. Scale bars=20 μm and nuclei are stained with DAPI (grey).

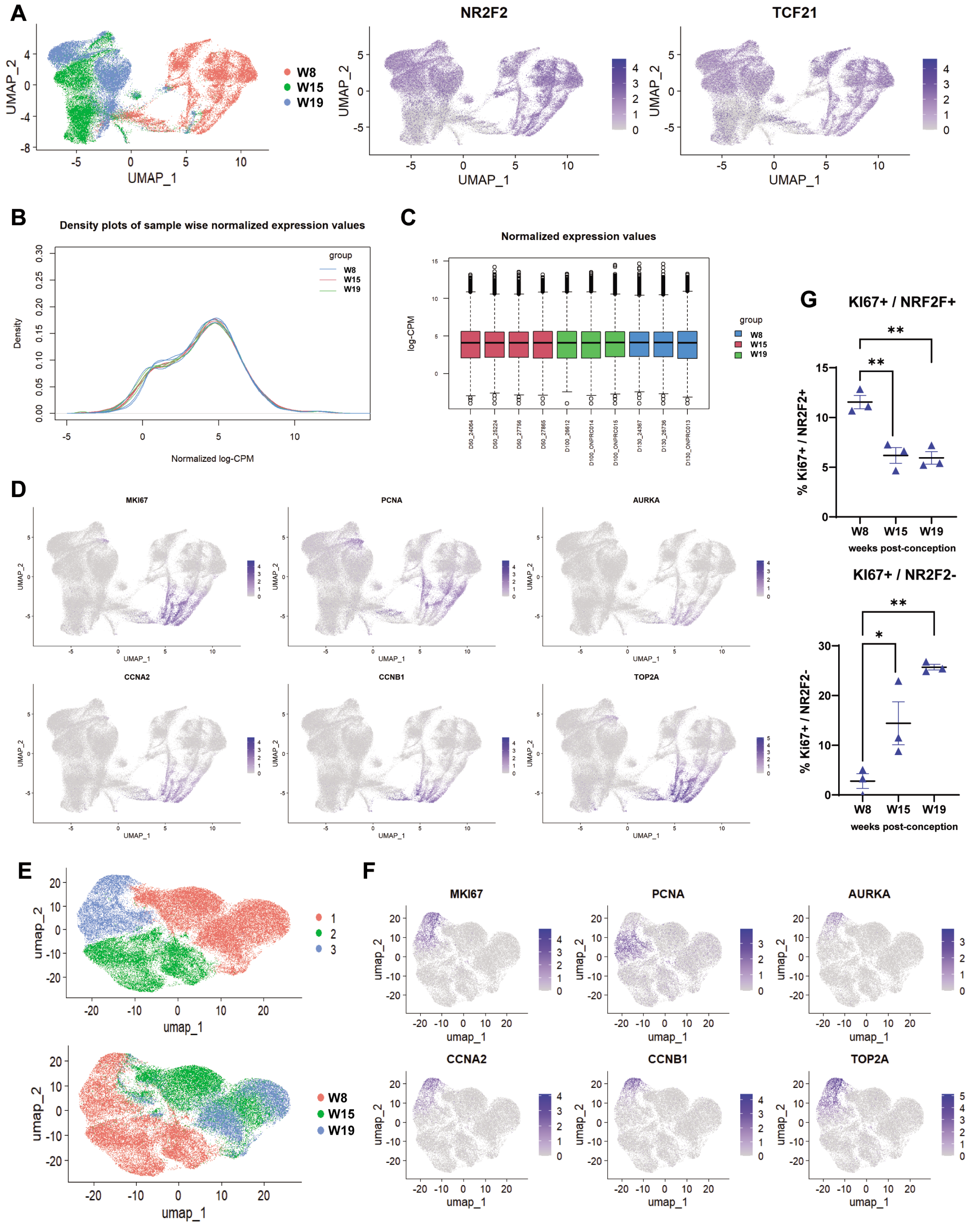

Fig. S5. 10X Genomics scRNA-seq analysis and proliferation of testicular interstitial cells. (A) UMAP plots for the age (right) and features of expression of *NR2F2* (middle) and *TCF21* (right), diagnostic markers of interstitial cells. **(B)** Density plot of normalized expression values for interstitial cells (Normalized log-CPM). **(C)** Graph of normalized raw counts (log-CPM) in the testicular somatic cell population defined in Fig. S3B using the trimmed mean of M values method (TMM). **(D)** UMAP from (A) displaying *MKI67*, a marker of cells in cycle, *PCNA* (a marker of cells in S-phase) and, *AURKA* (a Marker of cells in G2/M). These are enriched in W8 Interstitial cells as are the cell cycle regulators *CCNA2*, *CCNB1* and *TOP2A*. **(E)** UMAP plots for Interstitial Cells

sub-clustering showing three distinct sub-clusters (top), with cluster 1 and 2 correlating with W8 and cluster 3 with W15/W19 (bottom), respectively. **(F)** UMAP feature plots of *MKI67*, *PCNA*, *AURKA*, *CCNA2*, *CCNB1* and *TOP2A* gene expression in Interstitial Cells sub-clustering, with appears only in cluster 3 of W8. **(G)** Quantification of the % KI67-positive NR2F2-positive (top) or NR2F2-negative (bottom) detected in **Fig. 3I**. statistical differences were calculated using One-way ANOVA (significance was accepted at $p \leq 0.05$).

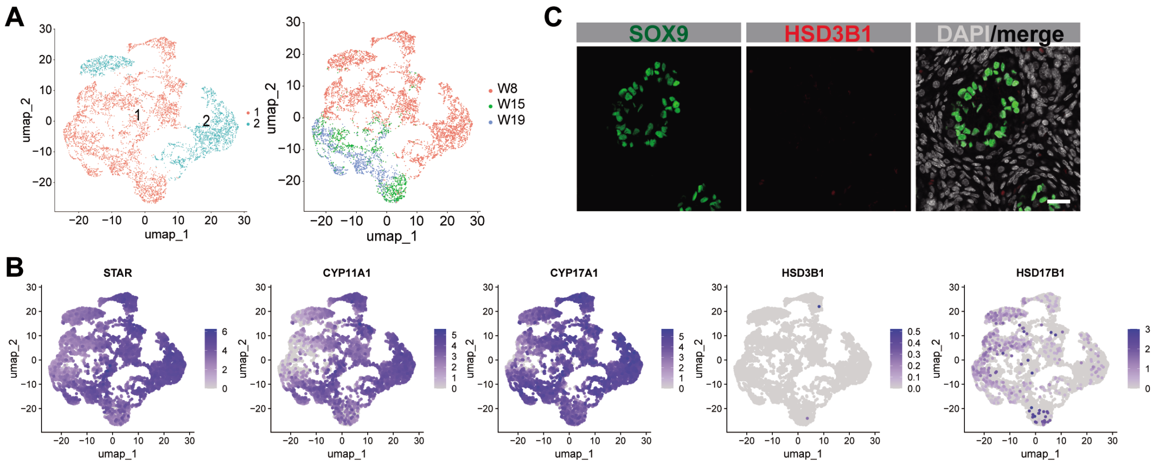

Fig. S6. Sub-clustering and testosterone-related enzyme gene expression in Fetal Leydig Cells. (A) UMAP plots for Fetal Leydig Cells sub-clustering showing two distinct sub-clusters (left), with cluster 2 only appearing in W8 (left). **(B)** UMAP feature plots of *STAR*, *CYP11A1*, *CYP17A1*, *HSD3B1* and *HSD17B1* gene expression in Fetal Leydig Cells sub-clustering. **(C)** IF for SOX9 (green) and HSD3B1 (red) at W19. Scale bar=20 μm and nuclei are stained with DAPI (grey).

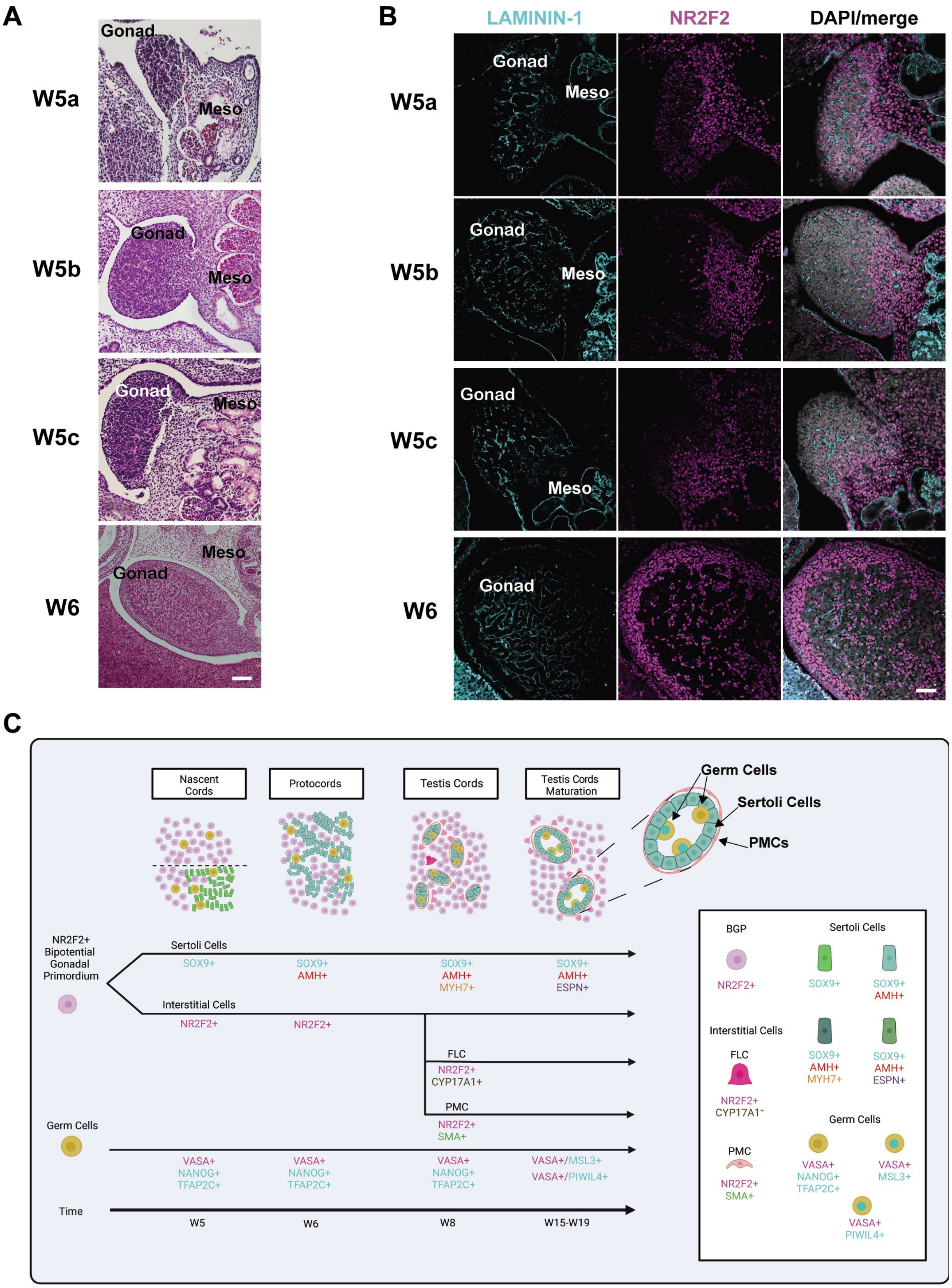

Fig. S7. Laminin Expression During Sex Determination and Gonadogenesis, and a Model of Rhesus macaque Embryonic and Fetal Testicular Development. (A) H&E-stained sections of Week (W)5a (indifferent), W5b (determined), W5c (determined), and W6 embryos with developing Gonad and Mesonephros labeled. **(B)** IF staining of W5a, W5b, W5c, and W6 for Laminin-1 and NR2F2. DAPI was used to stain for nuclei. Scale bars=50um. **(C)** Model

summarizing the specification and maturation of testicular cells during the 1st, 2nd, and 3rd trimesters. Bipotential Cells from the Gonadal Primordium (BGP) express NR2F2 prior to sex determination which occurs by W5 of embryonic development (1st trimester). At this timepoint Nascent cords start to form between Sertoli (SOX9+) and Interstitial (NR2F2+) and Germ (VASA+) cells. By W6 Protocords consisting of AMH+ Sertoli cells which have more contact with Germ cells, form into elongated and branched structures. At the same time, Interstitial cells are found forming the interstitial space between Protocords. By W8, around the end of the 1st trimester, Testis Cords have formed between Sertoli Cells and Germ cells with the appearance of Fetal Leydig Cells (FLC) which express both NR2F2 and CYP17A1 in the Interstitial space. Also, Peritubular Myoid Cells (PMC) cells co-expressing NR2F2 and SMA were found located in the interstitial space surrounding Sertoli cells, which express myosin heavy chain protein 7(MYH7). During the transition from the 2nd and 3rd trimesters (W15-W19) Testis cords are remodeled to form Testis Tubules structures containing germ cells positioned in either the lumen or basement membrane. Germ Cells (VASA+) shift from the expression of Primordial Germ cell (PGC) markers (NANOG/TFAP2C) and begin expressing the Fetal State 0 (Spermatogonial Stem Cell (SSC) precursor) marker PIWIL4. MSL3 is also expressed in Germ cells (VASA+) and is a new marker expressed during the period when Testis cords remodeling and maturation is also occurring.

Table S1. List of Animals time mated pregnant females used to collect testicles for analysis in this study

	Manuscript ID	Animal ID (Biological Replicates)	Sample ID	Assay performed
1.	W8_1	ONPRC002	W8_1A<br>W8_1B	10x genomics; IF
2.	W8_2	ONPRC009	W8_2A<br>W8_2B	10x genomics; IF
3.	W8_3	ONPRC010	W8_3A<br>W8_3B	10x genomics; IF
4.	W8_4	ONPRC011	W8_4A<br>W8_4B	10x genomics; IF
5.	W8_5	ONPRC026	W8_5A**<br>W8_5B**<br>W8_5C**	10x genomics; IF
6.	W15_1	ONPRC005	W15_1A*<br>W15_1B*	10x genomics; IF
7.	W15_2	ONPRC014	W15_2A<br>W15_2B	10x genomics; IF
8	W15_3	ONPRC015	W15_3A<br>W15_3B	10x genomics; IF
9.	W15_4	ONPRC023	W15_4A**<br>W15_4B**<br>W15_4C**	10x genomics; IF
10.	W19_1	ONPRC003	W19_1A<br>W19_1B	10x genomics; IF
11.	W19_2	ONPRC004	W19_2A<br>W19_2B	10x genomics; IF
12.	W19_3	ONPRC013	W19_3A<br>W19_3B	10x genomics; IF
13.	W5a	ONPRC022	CS16_1	IF
14.	W5b	ONPRC029	CS16_2	IF
15.	W5c	ONPRC033	CS16_5	IF
16.	W6	ONPRC019	CS20_1	IF

A and B refer to technical replicate of sample. * Sample was dissociated, frozen and then stored in liquid nitrogen prior to 10x genomics submission. ** Sample was obtained using the tubule enrichment (TubE) protocol.

Table S2. Quality Metrics of 10x data sets

	ID	# Raw reads	Mapability(%)	# Valid cells	# Genes/ cell	# UMIs/cell
1.	W8_1A	2.28×10^8	83.5	8,813	1,523	2,679
2.	W8_1B	2.36×10^8	83.8	6,077	1,981	3,788
3.	W8_2A	2.51×10^8	78.1	8,300	2,577	5,404
4.	W8_2B	2.58×10^8	78.0	7,471	2,778	6,037
5.	W8_3A	2.43×10^8	77.3	3,592	3,357	7,868
6.	W8_3B	2.32×10^8	78.3	5,704	2,690	5,713
7.	W8_4A	2.40×10^8	80.1	6,264	2,661	5,540
8.	W8_4B	2.58×10^8	80.0	5,462	2,974	6,811
9.	W8_5A	3.47×10^8	88.6	26,262	1,347	2,668
10.	W8_5B	3.00×10^8	88.8	25,611	1,314	2,584
11.	W8_5C	3.31×10^8	88.7	22,165	1,488	3,080
12.	W15_1A	2.08×10^8	77.7	1,708	2,040	4,122
13.	W15_1B	1.79×10^8	77.7	1,500	2,368	5,038
14.	W15_2A	2.58×10^8	74.1	5,488	2,068	4,375
15.	W15_2B	2.22×10^8	72.9	5,094	2,036	4,342
16.	W15_3A	2.13×10^8	76.2	4,066	2,083	4,604
17.	W15_3B	2.85×10^8	77.9	4,000	1,771	3,801
18.	W15_4A	8.13×10^7	83.5	12,554	646	1,072
19.	W15_4B	1.07×10^8	84.8	15,399	718	1,206
20.	W15_4C	1.26×10^8	84.0	15,951	757	1,279
21.	W19_1A	8.26×10^8	75.9	4,044	1,496	2,709
22.	W19_1B	7.96×10^8	76.4	4,466	1,668	3,162
23.	W19_2A	6.61×10^8	78.0	4,536	1,998	4,018
24.	W19_2B	6.47×10^8	77.6	5,421	1,916	3,806
25.	W19_3A	2.04×10^8	79.8	9,997	1,464	2,493
26.	W19_3B	2.21×10^8	78.8	7,999	1,650	2,976

Table S3. Primary Antibodies used for immunocytochemistry

Primary Antibody	Dilution	Company/ Cat #	RRID
Rabbit anti-NANOG	1:100	R&D Systems Cat# AF1997	RRID: AB_355097
Goat-anti-DDX4(VASA)	1:100	R&D Systems Cat # AF2030	RRID: AB_2277369
Rabbit-anti-DDX4(VASA)	1:100	Abcam Cat # ab13840	RRID: AB_443012
Mouse-anti-AP-2Y(TFAP2C)	1:200	Santa Cruz Biotechnology Cat # SC-12762	RRID: AB_667770
Rabbit-anti-PIWIL-4	1:100	Invitrogen Cat# PA5-31448	RRID: AB_2548922
Mouse-anti-MSL3	1:100	Abnova Cat# H00010943-B01P	RRID: AB_1114428
Mouse-Anti-Espin(E-7)	1:100	Santa Cruz Biotechnology Cat# SC-393469	RRID: AB_2905522
Rabbit-anti-SOX9	1:100	Millipore Cat # ab5535	RRID: AB_2239761
Rabbit-anti-Alpha-Smooth Muscle Actin (D4K9N)	1:100	Cell Signaling Technology Cat # 19245S	RRID: AB_2734735
Mouse-anti-COUP-TF II	1:200	Perseus Proteomics Cat# PP-H7147-00	RRID: AB_2314222
Rabbit-anti-LAMININ	1:200	Abcam Cat# Ab11575	RRID: AB_298179
Mouse-anti-hAMH	1:100	Bio-rad Cat# MCA2246	RRID: AB_2226471
Mouse-anti-CYP17A1	1:100`	Santa Cruz Biotechnology Cat# SC-374244	RRID: AB_10988393
Mouse-anti-hMYH7	1:100	R&D Systems MAB9096	RRID: AB_2915972
Mouse-anti-KI67	1:100	BD Pharmingen Cat 556003	RRID: AB_396287
Mouse-anti-Androgen Receptor (AR)	1:200	Biocare Medical ACI109A	
Rat-anti-GATA4	1:200	Thermo Fisher Scientific 14-9980-82	RRID:AB_763541
Mouse-anti-HSD3B1	1:200	Abcam ab55268	RRID:AB_942015

Table S4. Secondary Antibodies used for immunocytochemistry

Secondary Antibody	Dilution	Company Cat #	RRID
AF488-conjugated donkey-anti-mouse	1:200	Thermo Fisher A-21206	RRID: AB_2535792
AF488-conjugated donkey-anti-mouse	1:200	Thermo Fisher A-21206	RRID: AB_2535792
AF488-conjugated donkey-anti-rabbit	1:200	Life Technologies A-21131	RRID: AB_2535771
AF594-conjugated donkey-anti-mouse	1:200	Life Technologies A-21203	RRID: AB_141633
AF594-conjugated donkey-anti-rabbit	1:200	Jackson ImmunoResearch 711-585-152	RRID: AB_2340621
AF594-conjugated donkey-anti-goat	1:200	Jackson ImmunoResearch 705-586-147	RRID: AB_2340434
AF647-conjugated donkey-anti-mouse	1:200	Life Technologies A31571	RRID: AB_162542
AF647-conjugated donkey-anti-rabbit	1:200	Jackson ImmunoResearch 711-605-152	RRID: AB_2492288
AF647-conjugated donkey-anti-goat	1:200	Life Technologies A21447	RRID: AB_2535864
AF594-conjugated Donkey-anti-rat	1:200	Jackson ImmunoResearch 712-585-150	RRID:AB_2340688

Dataset S1 (separate file). DEG list of Sertoli Cells re-analysis.
(A) DE results of Pseudo-bulk analysis in Sertoli Cells
(B) The list of overlapped DEGs in two comparisons (W8 vs. W15 and W8 vs. W19)
(C) Specific marker results of sub-clustering in Sertoli Cells

Dataset S2 (separate file). DEG list of Interstitial Cells re-analysis.
(A) DE results of Pseudo-bulk analysis in Interstitial Cells
(B) Specific marker results of sub-clustering in Interstitial Cells

Dataset S3 (separate file). Gene list and GO/KEGG analysis of Fetal Leydig Cells sub-clustering.
(A) Specific marker results of sub-clustering in Fetal Leydig Cells
(B) GO and KEGG analysis of subcluster 2 in Fetal Leydig Cells

SI References

1. D. J. McCarthy, K. R. Campbell, A. T. Lun, Q. F. Wills, Scater: pre-processing, quality control, normalization and visualization of single-cell RNA-seq data in R. *Bioinformatics* **33**, 1179-1186 (2017).
2. Y. Hao *et al.*, Integrated analysis of multimodal single-cell data. *Cell* **184**, 3573-3587.e3529 (2021).
3. M. D. Robinson, A. Oshlack, A scaling normalization method for differential expression analysis of RNA-seq data. *Genome Biology* **11**, R25 (2010).
4. C. W. Law, Y. Chen, W. Shi, G. K. Smyth, voom: precision weights unlock linear model analysis tools for RNA-seq read counts. *Genome Biology* **15**, R29 (2014).
5. M. E. Ritchie *et al.*, limma powers differential expression analyses for RNA-sequencing and microarray studies. *Nucleic Acids Res* **43**, e47 (2015).
6. Y. Benjamini, Y. Hochberg, Controlling the False Discovery Rate: A Practical and Powerful Approach to Multiple Testing. *Journal of the Royal Statistical Society: Series B (Methodological)* **57**, 289-300 (2018).
7. J. Cao *et al.*, The single-cell transcriptional landscape of mammalian organogenesis. *Nature* **566**, 496-502 (2019).
